# Machine learning and phylogenetic analysis allow for predicting antibiotic resistance in *M. tuberculosis*

**DOI:** 10.1186/s12866-023-03147-7

**Published:** 2023-12-20

**Authors:** Alper Yurtseven, Sofia Buyanova, Amay Ajaykumar Agrawal, Olga O. Bochkareva, Olga V. Kalinina

**Affiliations:** 1 Department of Drug Bioinformatics, Helmholtz Institute for Pharmaceutical Research Saarland (HIPS), Helmholtz Centre for Infection Research (HZI), Campus E8.1, Saarbrücken, 66123 Saarland Germany; 2 Graduate School of Computer Science, Saarland University, Saarbrücken, 66123 Saarland Germany; 3 Institute of Science and Technology Austria (ISTA), Am Campus 1, Klosterneuburg, 3400 Austria; 4 Centre for Microbiology and Environmental Systems Science, Division of Computational System Biology, University of Vienna, Djerassiplatz 1 A, Wien, 1030 Austria; 5 Faculty of Medicine, Saarland University, Homburg, 66421 Saarland Germany

**Keywords:** Machine learning, Phylogeny, Antimicrobial resistance, Tuberculosis

## Abstract

**Background:**

Antimicrobial resistance (AMR) poses a significant global health threat, and an accurate prediction of bacterial resistance patterns is critical for effective treatment and control strategies. In recent years, machine learning (ML) approaches have emerged as powerful tools for analyzing large-scale bacterial AMR data. However, ML methods often ignore evolutionary relationships among bacterial strains, which can greatly impact performance of the ML methods, especially if resistance-associated features are attempted to be detected. Genome-wide association studies (GWAS) methods like linear mixed models accounts for the evolutionary relationships in bacteria, but they uncover only highly significant variants which have already been reported in literature.

**Results:**

In this work, we introduce a novel phylogeny-related parallelism score (PRPS), which measures whether a certain feature is correlated with the population structure of a set of samples. We demonstrate that PRPS can be used, in combination with SVM- and random forest-based models, to reduce the number of features in the analysis, while simultaneously increasing models’ performance. We applied our pipeline to publicly available AMR data from PATRIC database for *Mycobacterium tuberculosis* against six common antibiotics.

**Conclusions:**

Using our pipeline, we re-discovered known resistance-associated mutations as well as new candidate mutations which can be related to resistance and not previously reported in the literature. We demonstrated that taking into account phylogenetic relationships not only improves the model performance, but also yields more biologically relevant predicted most contributing resistance markers.

**Supplementary Information:**

The online version contains supplementary material available at 10.1186/s12866-023-03147-7.

## Introduction

*Mycobacterium tuberculosis* (Mtb), the causative agent of tuberculosis (TB), has been a major threat to public health for many years, and remains such a threat now. According to the World Health Organization (WHO), the estimated number of TB-caused deaths in 2021 alone was 1.6 million [1]. TB continues to pose a significant threat to global public health because of its ability to easily transmit and the occurrence of drug-resistant strains of Mtb. In 2019, WHO reposterd over ten million cases, including up to 4.5% of infections with drug resistant bacteria [1]. Early diagnosis and effective treatment are important steps in controlling the spread of TB and reducing its effect on public health. Given long cultivation time and complex phenotypic resistance testing of Mtb, screening for genetic markers of resistance presents an attractive alternative [2].

Known TB drugs act on Mtb via three mechanisms: first, by preventing the synthesis of the enzymes that makes up the cell wall; second, by interfering with ribosomes that affects protein production; and third, by inhibiting several DNA-level activities, including RNA/DNA synthesis [3]. Despite much research conducted on the subject, the drug resistance of Mtb is not fully understood, yet it is known that single nucleotide polymorphisms (SNPs) and other polymorphisms like insertions and deletions (INDELS) play a crucial role in that [4].

The increasing utilization of whole genome sequencing (WGS) for Mtb strains opens up new possibilities in identifying antimicrobial resistance. First, the phylogeny based methods such as phylogenetic convergence test and identification of genes under positive selection specific to resistant genomes were successfully applied for hundreds of genomes revealing genes and intergenic regions putatively responsible for resistance [5, 6]. Another commonly employed method for detecting significant resistance associated mutations in the data is genome-wide association study (GWAS). For bacteria, information about their population variations is primarily derived from sequences of their genomes. This, in combination with the fact that bacteria have very long genomic segments with strong linkage disequilibrium, creates a very specific setup for bacterial GWAS. This is aggravated by the presence of loci with multiple allelic variants and a large accessory genome (genes that are present only in some strains of a bacterial species, but not in others) [7]. Indeed, in Mtb recombination rates are particularly low [8], and thus virtually all loci of the genome are in linkage disequilibrium. On the other hand, the accessory genome of Mtb is very small, in contrast to other bacterial species, in which genes conferring resistance to particular antibiotics are often transferred via plasmids [9, 10].

Strong linkage disequilibrium between loci in bacteria implies that the population structure plays a major role and should be accounted for in GWAS studies. Indeed, many passenger mutations may be associated with a phenotype-relevant variant and will be called together with it, because they all step from a branch of closely related strains on the species’ phylogenetic tree. Such effects have been accounted for by using linear mixed models . In these models, the effect of each locus on the phenotype is modeled in the context of all other loci that are considered to contribute random effects. In this way, the effect of each locus that is strongly correlated with the background is systematically decreased. Linear mixed models showed promising results in bacterial GWAS for resistance phenotypes in many species including *E. coli*, *S. aureus*, *K. pneumoniae*, and Mtb [11]. Combination of GWAS approach with a phylogenetic convergence test in Mtb significantly improved the approach and allowed to identify epistatic interactions between drug-resistance-associated genes [12]. This approach is based on the idea that the true resistance-conferring mutations often originate at multiple branches of the phylogenetic tree of Mtb strains, while non-relevant passenger mutations occur in single (but maybe very populated) branches. Such effects are not visible when the strains are considered to be independent as in classical GWAS, but may be accounted for when population structure is taken into account.

Apart from population structure and epistasis, other factors like recombination rate, within-host diversity, polygenicity and multi-allelic SNPs also need to be taken into account while performing the GWAS in bacteria. Various computational tools and methods have been developed to account for these factors [13]. For example, CCTSWEEP [14], VENN [14] and GWAMAR [15], use phylogenetic trees to account for population structure, but do not take other factors into account. Other phylogenetic tree based methods like treeWAS [16] takes all factors into account except within-host diversity, while Scoary [17] does not consider within-host diversity as well as recombination rate. Among all the available bacterial GWAS tools, SEER [18] and pyseer (python implementation of SEER) [19] are the only two that considers most of the pitfalls one can stumble upon in bacterial GWAS. Both the tools use linear models with fixed or mixed effects to perform the GWAS studies. Although the linear mixed models are the best performing models in GWAS studies, they are not well suited for detecting interactive and non-linear effects. In such cases, previous studies have used various machine learning approaches like random forests, gradient boosting, neural networks etc. as they can perform significant attribute selection, can identify complex interactions between attributes and can also capture non-linear interaction of SNPs [20–22]

Over the last few years, certain rule-based approaches like TB-Profiler [23] have been developed to detect the phenotype of newly sequenced Mtb strains. These approaches work by calling out the variants and comparing them against curated databases. Thus, these approaches can only detect resistance caused by known markers. To overcome this, prediction approaches based on machine learning (ML) have been recently explored for the identification of resistance associated mutations in bacteria. Similar to GWAS, the resulting population structure can be a significant confounder in the ML models as well. To account for population structure, 414 strains of *P. aeruginosa* have been investigated with a host of classical machine-learning techniques by employing a training strategy based on blocks of phylogenetically related sequence [24]. In another study which used 1681 *E.coli* strains for predicting AMR, they generated the population structure matrix based on core genome alignment of strains and showed that the performance of their ML models improved when accounted for population structure [25]

In recent years, various studies have been published studying drug resistance specifically in Mtb. Zhang et. al used dN/dS ratio (the ratio of non-synonymous to synonymous SNPs) to identify important genes and SNPs related to Mtb resistance. They showed that apart from SNPs in coding regions, SNPs in intergenic regions are also strongly correlated to resistance in Mtb [5]. Other approaches for detecting resistance in Mtb employed different ML models and achieved area under ROC curve values up to 0.95 in a classification task for resistance towards selected drugs using features from 23 selected target genes known to be implicated in resistance development [26, 27]. A recent computational framework, TB-ML, provides implementations for different ML methods such are random forest, direct association and convolutional neural networks [28]. Treesist-TB, a customized decision tree-based machine learning algorithm for predicting resistance in Mtb, aims to extract genomic variants which might have been missed because of overfitting problems of the standard machine learning algorithms [29]. Furthermore, since resistance to multiple drugs (multidrug resistance) is possible in bacteria, multi-label ML methods have been utilized to predict resistance and to detect novel resistance associated mutations [30, 31]. Besides traditional machine learning, different deep learning approaches have also been applied to predict the resistance in bacteria. DeepAMR is one such method that integrates deep denoising auto-encoder and multi-label classification into an end-to-end model with added explainability to models [32]. AMR-Diag is another example of a deep learning based method that uses assembly-free neural network for predicting phenotypic resistance of *E.coli* and *K.pneumoniae* towards 3rd generation cephalosporins and carbapenems [33]. Training datasets for these methods can be found in public resources, such as, for example, the PATRIC database [34].

However, to date most models trained with ML algorithms do not account for specifics of the bacteria data, such as population structure and linkage disequilibrium, and thus can be prone to misinterpretation. Therefore, just as for GWAS studies, ranking genetic variants is a crucial part that should be conducted before applying algorithms for model training to filter out non-significant features from training dataset. One of suggested ways to rank such variants is to predict their potential impact on protein function [35]. An indirect way to take population structure into account is to design the training process specifically for each set of bacterial genomes by splitting the isolates into training, test, and validation sets based on genomic distance between them [36].

Quantification of the phylogenetic signal of genotypic traits also might be used for variants ranking based on the population structure. Originally phylogenetic signal indices, such as Pagel’s $\lambda$, Blomberg’s *K*, Moran’s *I*, Abouheif’s $C_{mean}$, were developed for molecular ecology questions, where non-independence of traits was used to seek evidence for adaptation in the patterns of correlated trait evolution (such as size, shape, life history and behavior) across contemporary species [37, 38]. If the phylogenetic signal index of a particular trait is calculated, it can be compared with values expected under random traits distribution which can be generated analytically or be numerically simulated by random permutations to test the null hypothesis of no phylogenetic signal. Analytically random traits distribution for continuous characters is traditionally generated under a Brownian motion (BM) model, which assumes random walk along the branches of the phylogenetic tree, with the variance in the distribution of trait values being directly proportional to branch length [39]. Note that these indices respond differently to inaccuracies in phylogenetic tree construction, absence of branch length information and low sample size [40].

As many variables analyzed in comparative genomics are binary, several approaches for estimation of phylogenetic signal in a binary trait were later also developed [41–43]. In particular, Fritz et al. suggested D-score, a measure based on the sum of sister-clade differences in a given phylogeny [43]. While developed to predict extinction risk for species or clades of unknown risk status, it is still widely used to answer different ecological questions [44, 45]. Note that this statistic is sensitive to inaccuracies in tree topology and requires tree rooting, thus it is not suitable for short-scale bacterial phylogenetic trees which are often affected by homologous recombination and horizontal gene transfer [46]. Recently we developed an alternative approach to estimate the discordance of genomic features with phylogeny in a bacterial population [47]. To rank the binary traits based on their independence from population structure, we first performed the ancestral reconstruction of trait states across the phylogenetic tree and then estimated the inconsistency based on the number of state changes and the phylogenetic distances between nodes where it happened. This measure, called parallelism score, is less sensitive to tree rooting and inconsistencies in clades with short branches, which is often the case while reconstructing phylogeny of closely-related bacterial species.

In this study, we present a novel phylogeny-based method for ranking genetic variants followed by training ML models for predicting antibiotic resistance in Mtb. We demonstrate that this filtering is crucial and improves performance of the ML models. Using bacterial GWAS methods as a baseline, we identified known resistance-associated variants in a set of Mtb strains with known resistance profiles from the PATRIC database, as well as detect novel potential resistance-associated variants.

## Results

**Fig. 1:**
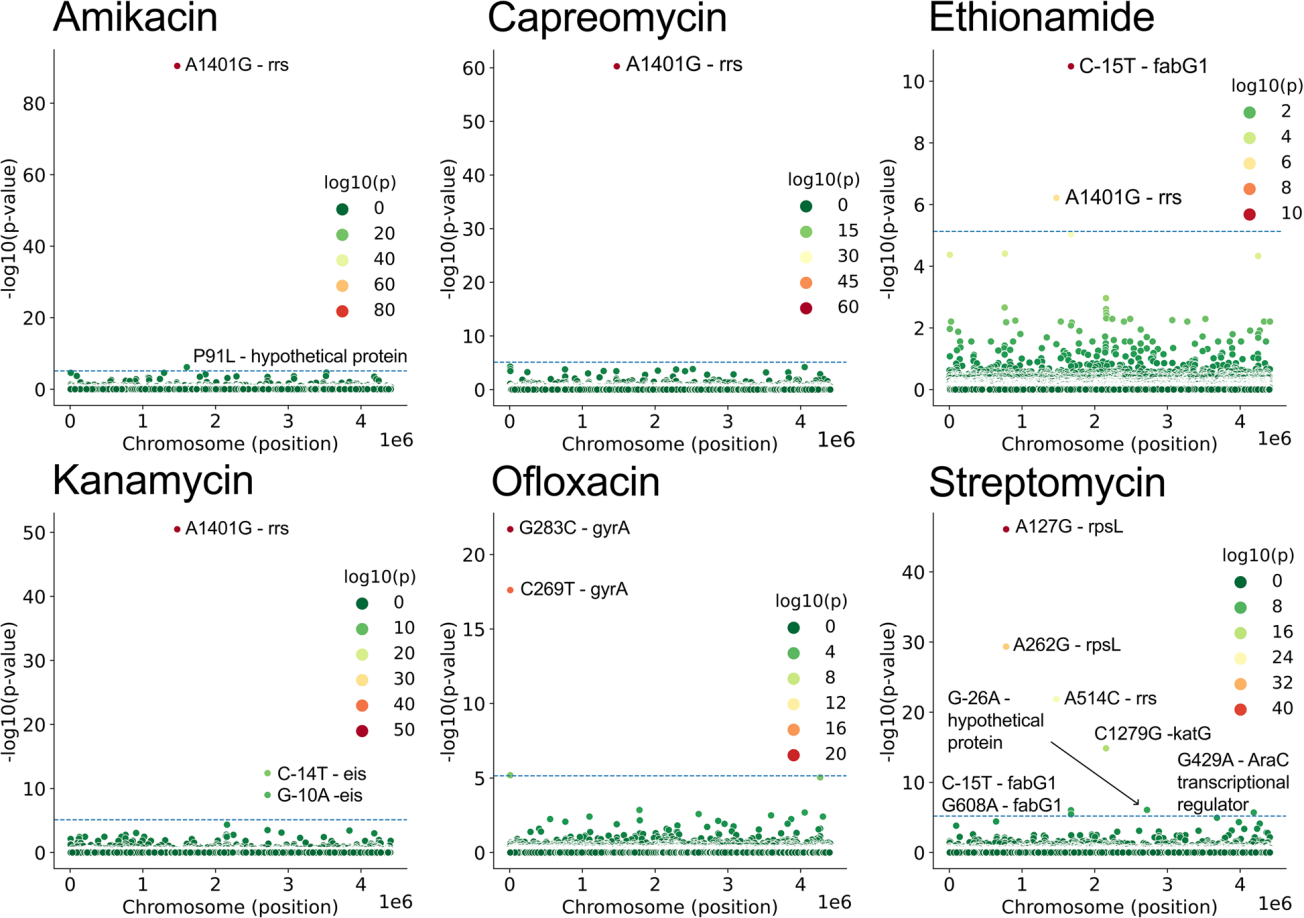
Associations between SNP and antibiotic resistance found using GWAS (see Methods). Horizontal line shows selected threshold for significance

First, we established a baseline with GWAS analysis. The pyseer software identified key known resistance mechanisms for the corresponding antibiotics (Fig. 1, Supp. Table S2): mutations in 16S ribosomal RNA gene for aminoglycosides, mutations in 30S ribosomal protein S12, catalase peroxidase *katG*, and 3-oxoacyl-ACP reductase *fabG* genes for streptomycin, SNPs upstream of the aminoglycoside acetyltransferase *eis* gene for kanamycin, and mutations in DNA gyrase subunit A for ofloxacin. We did not observe any mutations known to confer resistance to ethionamide; instead for this drug, which is a second-line therapyone, we observed a mutation in the 16S ribosomal RNA gene, which may be an indication of multi-resistance against first-line streptomycin or other aminoglycosides. In addition, we observed several unreported mutations in and upstream of genes which encode hypothetical proteins and a transcription regulator from the AraX/XylS family that have less significant *p*-values.

**Fig. 2:**
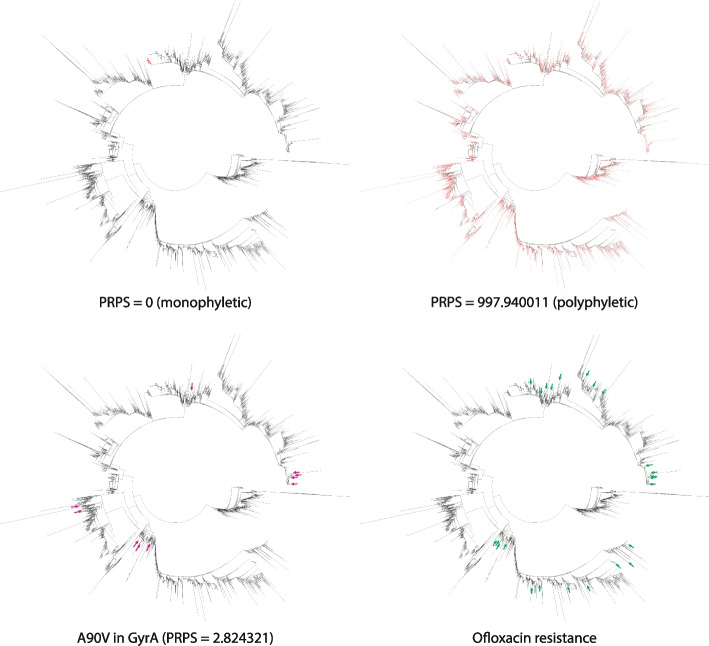
Top: High- and low-PRPS SNPs. Branches corresponding to the strains containing the SNP are colored red. Bottom: Mutation A90V in GyrA associated with resistance to fluoroquinolones (left) and ofloxacin-resistant strains (right). Branches corresponding to strains with the mutation A90V in GyrA are colored magenta and indicated with arrows. Branches corresponding to ofloxacin-resistant strains are colored green, and the corresponding clades are marked with green arrows

In the ML analysis, first we calculate phylogeny-related parallelism score (PRPS), a measure of inconsistency between SNPs phylogenetic pattern and the species tree topology, to exclude mutations that are strongly linked with the population structure from the training dataset (see Methods). According to our procedure, SNPs whose distribution is consistent with phylogenetic tree structure have low PRPS. In contrast, high PRPS indicate independent acquisition of SNPs by different lineages. PRPS reflects whether a SNP is monophyletic or polyphyletic, where high PRPS scores correspond to highly polyphyletic features (Fig. 2, top). For comparison, a known resistance-associated mutation A90V in GyrA that confers a strong resistance to fluoroquinolones [48], has a PRPS score of 2.824321 and is in the top 11% of the PRPS-ranked feature list (Fig. 2, bottom).

Top 30% of features with highest PRPS were used for training our ML models. In all cases, even though a significant number of mutations (70%) were not considered in the models, both MCC and ROCAUC values increase in almost all cases when high-PRPS mutations are used as features (the only exception being the RF models for amikacin, where the performance is almost perfect anyway). To test the significance of this observation, we randomly deleted 70% of the mutations from our data and re-trained the models. In this case, all performance measures dropped drastically, which proved that SNPs selected by the phylogeny analysis were not random and were enriched in predictive features, while containing less noise (Table 1). Moreover, using high-PRPS features increased the training speed and reduced the amount of resources used for model training in terms of memory and CPU time. Additionally, we checked for presence of known antibiotics resistance markers among the predictive features using features importance analysis, and in all cases these markers are retained in the high-PRPS models and disappear in random-30% models (data not shown). Thus, we used top 30% high-PRPS features for all further analysis.

**Table 1:** Comparison of different feature sets

	Support Vector Machine (SVM)			Random Forest		
	All features	30% PRPS	30% Random	All features	30% PRPS	30% Random
MCC						
AMK	0.720	0.752	0.302	0.881	0.883	0.481
CAP	0.539	0.620	0.334	0.779	0.780	0.569
ETH	0.325	0.370	0.269	0.550	0.605	0.488
KAN	0.766	0.685	0.415	0.812	0.856	0.546
OFL	0.508	0.549	0.294	0.778	0.778	0.452
STR	0.602	0.613	0.477	0.782	0.801	0.650
ROCAUC						
AMK	0.842	0.872	0.663	0.927	0.918	0.765
CAP	0.779	0.816	0.683	0.858	0.863	0.718
ETH	0.665	0.686	0.640	0.780	0.807	0.746
KAN	0.873	0.844	0.718	0.880	0.916	0.741
OFL	0.757	0.781	0.649	0.870	0.870	0.723
STR	0.798	0.816	0.747	0.865	0.890	0.785
Sensitivity						
AMK	0.721	0.786	0.485	0.868	0.843	0.671
CAP	0.650	0.720	0.517	0.733	0.760	0.453
ETH	0.603	0.603	0.676	0.689	0.779	0.691
KAN	0.787	0.755	0.585	0.777	0.681	0.532
OFL	0.719	0.771	0.573	0.771	0.771	0.625
STR	0.687	0.741	0.642	0.752	0.815	0.601
Specificity						
AMK	0.965	0.958	0.840	0.987	0.994	0.858
CAP	0.910	0.912	0.849	0.983	0.979	0.982
ETH	0.727	0.769	0.603	0.810	0.835	0.802
KAN	0.961	0.933	0.851	0.983	0.992	0.949
OFL	0.796	0.790	0.725	0.970	0.970	0.820
STR	0.910	0.891	0.853	0.977	0.965	0.969

We further investigated our SVM and RF models with permutation-based feature importance analysis [49] to identify features that are most contributing to prediction outcome and thus should be investigated for being causative variants for resistance mechanisms (Supplement 3 Tables 1 & 2). First, with our ML approach we identified a list of common known resistance-associated mutations. For example, missense variants in the DNA gyrase gene *gyrA* are known to cause ofloxacin resistance, and mutations in the 16S ribosomal RNA gene *rrs* and small ribosomal subunit protein gene *rpsL* are causative for aminoglycosides resistance. Futhermore, mutations in the catalase-peroxidase gene *katG* that are known to cause resistance to isoniazid and prothionamide, and in the arabinosyltransferase A gene *embA* cause resistance to ethambutol. In several cases we observed SNPs upstream of known resistance-associated genes, which may signify the importance of regulation of gene expression in resistance, also reported in literature [50–52]. In particular, we identified -15C>T (15 bases upstream of the start codon) mutation in the *fabG1* gene that is known to be associated with ethionamide resistance and -165T>C mutation in *rpsL* gene that is associated with aminoglycoside resistance. Moreover, we observed new connections of known resistance markers with other antibiotics. For example, *katG* is known to be associated with resistance to isoniazid and prothionamide [53, 54], which are a first and second-line drugs respectively, but we see a Arg463Leu mutation in *katG* to be predictive of resistance to streptomycin. In addition to known resistance-associated variants, we identified previously undescribed variant resulting in non-synonymous mutation: Ile145Met in a probable amino acid aminotransferase PabC.

To investigate the robustness of our models, we also have trained models with different seeds for random split (see Methods). 10 randomly selected seeds have been used, and they all yielded similar results both for model’s MCC (Matthews correlation coefficient) and AUROC (Area under the receiver operating characteristic) values, as well as permutation importance feature analysis. We have observed higher MCC and AUROC values for random forest as compared to SVM for every antibiotic (Table 2).

**Table 2:** Performance of trained ML models with random seeds

	Support Vector Machine (SVM)				Random Forest			
	Hold-out		Cross Validation		Hold-out		Cross Validation	
	MEAN	STDEV	MEAN	STDEV	MEAN	STDEV	MEAN	STDEV
MCC								
AMK	0.775	0.034	0.771	0.057	0.859	0.026	0.853	0.059
CAP	0.671	0.061	0.661	0.060	0.766	0.035	0.799	0.040
ETH	0.367	0.072	0.353	0.068	0.519	0.054	0.566	0.074
KAN	0.688	0.031	0.686	0.047	0.820	0.044	0.866	0.025
OFL	0.555	0.037	0.571	0.068	0.801	0.035	0.809	0.024
STR	0.626	0.026	0.611	0.030	0.799	0.013	0.816	0.018
ROCAUC								
AMK	0.873	0.021	0.874	0.031	0.904	0.027	0.895	0.042
CAP	0.843	0.028	0.849	0.034	0.865	0.012	0.885	0.023
ETH	0.685	0.039	0.677	0.036	0.760	0.027	0.777	0.031
KAN	0.850	0.021	0.853	0.033	0.889	0.023	0.919	0.019
OFL	0.780	0.018	0.786	0.032	0.887	0.015	0.891	0.014
STR	0.821	0.013	0.815	0.013	0.892	0.007	0.898	0.013

To predict the impact of non-synonymous coding variants on the proteins’ function we analyzed their location in the protein three-dimensional structure with StructMAn [55]. This tool predicts location of mutated position with respect to potential binding interfaces as well as protein core based on analysis of all experimentally resolved structures of complexes of homologous proteins. Thus, even if a certain binding event has never been detected for a particular bacterium, we can hypothesize about them by transferring information from related species. Most known resistance-associated mutations as well as previously undescribed variants can be mapped onto a structure where they lie on an interaction interface or in the protein core. For some new variants, there are neither any experimentally resolved structures of the corresponding protein, nor of its homologs. In this case we relied on structures predicted with AlphaFold and four out of six of these variants are classified as surface. No complexes of these proteins have been resolved, so it is possible that these surface mutations in fact take part in some biologically important interactions (Table 3).

**Table 3:** Structural classification of known resistance-associated and novel predictive variants

Variant ID	Gene	Mutation	Uniprot ID	RIN-based simple classification	Observed resistance to	Reported resistance to
Known resistance-associated mutations						
(7570, ’C,T’, ’snp’)	*gyrA*	A90V	P9WG47	Ligand interaction	OFL, ETH	FQ [53, 54]
(7362, ’G,C’, ’snp’)	*gyrA*	E21Q	P9WG47	Protein interaction	OFL	FQ [53, 54]
(781687, ’A,G’, ’snp’)	*rpsL*	K43R	P9WH63	RNA interaction	STR	STR [53, 54]
(2154724, ’C,A’, ’snp’)	*katG*	R463L	P9WIE5	Surface	STR	INH, PTO [53, 54]
Previously unreported variants						
(781395, ’T,C’, ’snp’)	*rpsL*	T-164C	P9WH63	-	STR	-
(906857, ’A,G’, ’snp’)	*pabC*	I145M	Q79FW0	Protein interaction	ETH	-
Previously unreported variants with no structural templates (AlphaFold [56] models used)						
(1670814, ’C,T’, ’snp’)	*DUF58*	G134G	A0A7U4G1E6	Core	ETH	-

**Fig. 3:**
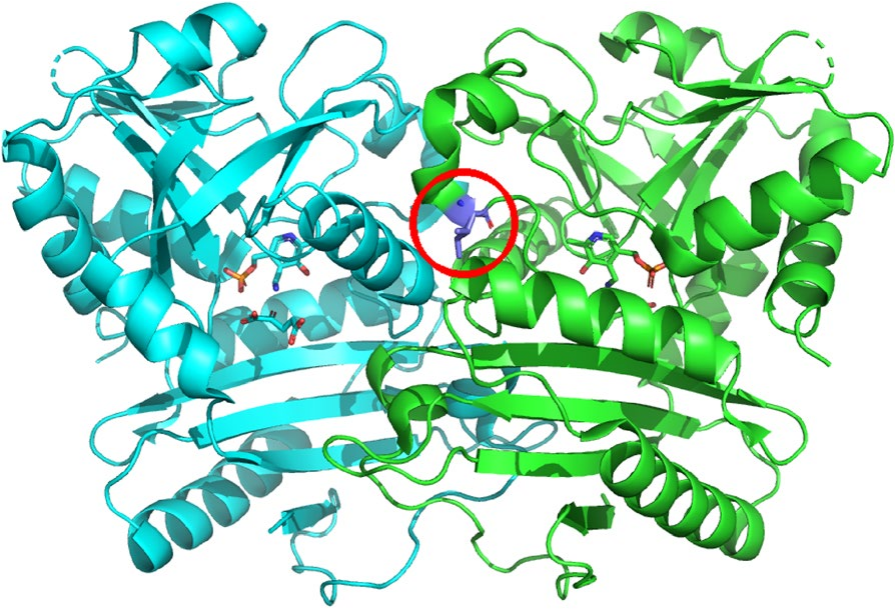
Structural analysis of the novel potential resistance marker. Corresponding mutation is shown as violet sticks, different chains in multimeric protein complex is shown with ribbons in different colors. Figure was created with PyMOL [57]. Mutation Ile145Met in the putative amino acid aminotransferase PabC, mapped on 3D structure, PDB id 6Q1S

For example, mutation Ile145Met in the putative amino acid aminotransferase PabC (corresponds to SNP 906857A>G) is located on a protein-protein interaction interface of the protein (Fig. 3). Although the role of this protein in ethionamide resistance is not clear, this mutation may change affinity of interaction between the subunits, and thus impact protein function.

## Discussion

Large collections of M. tuberculosis genomes provide a basis to study genomic features that cause antibiotic resistance in their populations. Understanding of molecular mechanisms, how both classical and new antitubercular drugs work and identifying specific mutations that allow Mtb to escape the effects of these drugs are important for development of new extended diagnostic panels as well as new efficient methods of treatments.

Traditional pipelines for Mtb drug resistance are based on calling known resistance-associated mutations in the newly sequenced genomic data (direct association), and work quite well with nearly perfect specificity. Meanwhile, ML approaches can improve this performance further [26]. However, this study focussed only on 23 genes known to be associated with drug resistance, considering only on a small number of arguably most relevant features, and thus missing the possibility to discover novel resistance mechanisms. ML methods show promise not only for Mtb drug resistance, but for work on other pathogens as well [24], slowly making their way into personalized clinical guidelines [58].

In this work we propose a set of ML models for predicting antibiotics resistance in Mtb trained on public data. Despite the fact that our models are agnostic of prior biological knowledge on resistance markers and mechanisms, they were able to re-discover many of these known resistance markers. In addition to that, we have observed previously unreported resistance-associated mutations: For instance, we detected markers known to be associated with resistance to other antibiotics than those that were used in the phenotypic screens under consideration: for example, in the data for streptomycin (first-line drug) we detected a mutation in *katG* that is associated with resistance to a first and second-line drugs isoniazid and prothionamide [53, 54], which may hint at multi-resistance. This emphasizes the importance of using whole-genome sequences in such models as they are a mine of additional information.

Using whole-genome sequences, however, is connected with a danger of discovering many false-positive associations, due to low recombination rates and strong population structure in bacteria [11]. Different approaches have been suggested to account for this: using linear mixed models [11], adding a feature that reflects predicted functional impact of the genetic variant [35], or training while accounting for population structure in the sample [24]. We proposed a simple procedure and measure (parallelism-related phylogenetic score, PRPS) to rank genetic variants by their propensity to occur at multiple locations within the species’ phylogenetic tree and show that using only top-PRPS features improves performance of the models and minimizes the number of potential false positives. Combinations of multiple such scores are thinkable: for example, with predicted functional impact of corresponding mutations [59–61] or with their potential impact on protein structure and interactions [55].

We have demonstrated the potential of ML methods and compared them to the state-of-the-art GWAS approaches. In a certain sense, the way an ML algorithm combines the effects of different features is similar to how polygenic risk scores (which are also used in GWAS, but primarily in connection with human disease [62]) combine the effect of different mutations. Whereas classical PRS combine the effects linearly, ML approaches are finding their way in more advanced versions of PRS to capture non-linear (epistatic) effects [62–64].

ML methods are not restricted to SNPs. Whereas SNPs play a major role for resistance development in Mtb, other mechanisms are prevalent in other pathogens, such as horizontal gene transfer of resistance-associated genes via plasmids. Accordingly, features related to gene presence can be easily incorporated into models. Thanks to advances and increasing accessibility of long-read sequencing technologies, other potential mechanisms can be included and their impact on resistance can be explored: gene copy number, chromosomal rearrangements etc. [65–67]. Whereas sequencing becomes cheaper, phenotypic screens that are required to train ML models are still laborious and costly, hence algorithmic developments to make most out of limited data are essential, too.

## Conclusion

We present ML models for the discovery of antibiotic resistance markers in Mtb. The models are trained using whole-genome sequences with accompanying resistance screens, and the resistance markers are extracted with feature importance analysis. We emphasize the importance of accounting for population structure within a bacterial species by introducing PRPS, phylogeny-related parallelism score. We show that ML models that employ PRPS-aware features demonstrate superior performance, as well as discover more biologically meaningful markers. Additionally, we show that it is possible to uncover markers related to first-line drugs when analyzing screens for second-line drugs, as multi-resistance occurs quite frequently. We report several new potential resistance markers and discuss the corresponding possible molecular mechanisms.

## Methods

### Genomic and phenotypic data and variant calling

Genome sequences and the resistant phenotype data of the Mtb strains for six antibiotics (amikacin, capreomycin, ethionamide, kanamycin, ofloxacin and streptomycin) were downloaded from PATRIC database [34] (retrieved on November 26, 2021). First, the strains were filtered out because the corresponding fasta files were corrupted in the database. Next, the duplicate genomes and the cases when identical genomes were annotated with conflicting phenotypes were further removed. Additionally, genomes with more than 5 consecutive ‘N’ nucleotides, L90 > 100 and those comprising more than 999 contigs were excluded from our study. To get rid of contamination, all genomes with maximum pairwise Mash genetic distance exceeding 0.2 to any other genome were removed. Finally, genomes with the length deviating by more than two standard deviations from the average Mtb genome length and those with more than 5% contamination were excluded from our study. After applying these filters, our final dataset consisted of 4869 genomes (Supplement 4 Table 2) which were used in our study (Table 4).

**Table 4:** Datasets used in this study. For the number of strains and SNPs, the final numbers after all filtering are provided

Drug name	Line of therapy	Pharmacological group	Number of strains	Number (fraction) of resistant strains	Number of SNPs (features)
STR	First line	Aminoglycosides	4,726	1158 (24,5%)	24,425
AMK	Second line	Aminoglycosides	1,149	208 (18,1%)	18,864
CAP	Second line	Aminoglycosides	1,086	205 (18,9%)	17,045
KAN	Second line	Aminoglycosides	1,362	297 (21,8%)	17,335
OFL	Second line	Fluoroquinolones	795	307 (38,6%)	14,185
ETH	Second line	Nicotinamide derivative	571	210 (36,8%)	12,974
Total			4,869		24,425

Whole genome sequences of all the 4869 strains were mapped to the H37Rv reference genome (NCBI accession: NC_000962) and variant calling was performed using the Snippy [68]. Variants present in less than 0.2% strains were filtered out to account for sequencing errors. Among the variants, we removed INDELS and only retained single-nucleotide polymorphism (SNPs). Finally, we were left with 24,425 SNPs which were then used as a features for further downstream studies.

### Genome wide association studies (GWAS)

As a baseline, genome wide association analysis was carried out using pyseer [19], since this tool accounts for most of the pitfalls that one can come across in bacterial GWAS [13]. To account for strong population structure, a similarity kinship matrix was generated using “similarity_pyseer” command of pyseer and was used as input for GWAS analysis. Further, the linear mixed models (LMM) which models other SNPs as random effects to control for population structure was used to perform the GWAS analysis. Finally, to control for multiple testing, the Bonferroni correction was used, and a *p*-value threshold of 0.05 divided by number of variants was adopted for all antibiotics to select the significant variants associated with resistance phenotype.

### Phylogenetic reconstruction and phylogeny-related parallelism score

To build the phylogeny of the Mtb strains, we used the PanACoTA pipeline [69]. Orthologous groups were constructed with 80% threshold for protein identity, concatenated codon alignment of 161 single-copies common genes was used to construct the maximum-likelihood phylogenetic tree using fasttree [70] with 1000 bootstrap replicates. The phylogenetic tree with resistance profiles was visualized using the iTOL online tool [71]. To calculate the phylogeny-related parallelism score (PRPS) for each SNP we perform the following procedure (adopted from [72]). First we generate a matrix of the pairwise distances between all the nodes in the tree. Then we collapse the clades where all leaves have a SNP and assign this SNP to the corresponding ancestral node. Finally we calculate the PRPS as a logarithm of a sum of pairwise distances between the nodes with SNP:

$$\begin{aligned} PRPS_{SNP} = log\left( \sum\nolimits_{i,j\epsilon N i\ne j} D\left( N_{i}, N_{j} \right) \right) \end{aligned}$$

where N are nodes which the SNP is assigned to, D is the phylogenetic distance between them. Thus, PRPS reflects the number of occurrences of a SNP across the tree and distances between the nodes where it occurred.

### Machine learning for predicting antimicrobial resistance

Resistance profiles from the PATRIC database were used as the target variable for machine learning methods. To this end, the minimal inhibitory concentration (MIC) values were converted into a binary variable representing ‘resistant’ and ‘susceptible’ phenotypes. We used two supervised learning models; support vector machine (SVM) with linear kernels and random forest (RF) algorithms. For SVM, data were divided into training and test sets randomly with 67% and 33% going into the training and test sets, respectively. Models were trained on the training set with the hold-out validation technique with different C values and tested on the test set. Best models were selected by choosing the ones with the highest MCC (Matthews correlation coefficient) score. To validate robustness of the models, we have trained additional 20 models with different randomization seeds with 30% top PRPS score features. We used the hold-out strategy with 67% training and 33% test split (10 models), and 4-fold cross validation with 80% going to training/validation and 20% to the test set (10 models). Training and validation sets were used to optimize for different C values. For RF, the whole training procedure was analogous to SVMs. Hyperparameters were optimized with Auto-sklearn 2.0 [73] using the hold-out resampling strategy. Again, we trained additional ML models with different randomization seeds and applied hold-out and cross-validation strategies. All ML models were implemented with scikit-learn [74] and auto-sklearn [73] in Python.

Three feature sets were used for our ML models: (1) all SNPs selected as described above; (2) 30% features with highest PRPS; (3) 30% features chosen randomly from all features. The 30% threshold was chosen, since it consistently delivered best performance for the PRPS-ranked features (data not shown). Further, feature importance analysis was performed to extract predictive resistance genetic markers. For RF models, we used permutation-based feature importance analysis [74] with significance threshold of $r^2 > 0.03$. For SVMs we used both permutation-based feature importance analysis with significance threshold of $r^2 > 0.03$ and coefficients. For additional models, important features for each model are calculated by using permutation-based feature importance analysis [74]. For each model, important features were collected with $r^2 > 0.03$. For each antibiotic and model technique, a feature is deemed important if it was observed as important in at least 8 out of 10 corresponding randomized models.

### Supplementary Information

**Additional file 1.**

## Data Availability

Implementation of the models, trained models and data tables can be found on GitHub: https://github.com/AlperYurtseven/ML-PRPS-MTB.

## Abbreviations

AMK: Amikacin
AMR: Antimicrobial resistance
CAP: Capreomycin
EMB: Ethambutol
ETH: Ethionamide
FQ: Fluoroquinolone
GWAS: Genome-Wide Association Study
INH: Isoniazid
KAN: Kanamycin
ML: Machine Learning
OFL: Ofloxacin
PTO: Prothionamide
RF: Random Forest
STR: Streptomycin
SVM: Support Vector Machine
