# Supplementary material for: Machine learning and phylogenetic analysis allow for predicting antibiotic resistance in M. tuberculosis
Source: BMC Microbiol. 2023 Dec 20;23:404. doi: 10.1186/s12866-023-03147-7 (PMC10731705; doi:10.1186/s12866-023-03147-7)
Supplement: Supplementary file 1 — Additional file 1. [file 12866_2023_3147_MOESM1_ESM.zip › Supplement_1.pdf]

Supplement 1

Table 1: Genes associated with antibiotic resistance to different drugs from previous studies

**Drug name**	**Line of therapy**	**Pharmacological group**	**Gene name**	**References**
Streptomycin	First line	Aminoglycosides	gidB, rrs, tlyA, rpsL	(53, 54)
Amikacin	Second line	Aminoglycosides	gidB, rrs, tlyA	(53, 54)
Capreomycin	Second line	Aminoglycosides	gidB, rrs, tlyA	(53, 54)
Kanamycin	Second line	Aminoglycosides	gidB, rrs, tlyA, eis	(53, 54)
Ofloxacin	Second line	Fluoroquinolones	gyrA, gyrB	(53, 54)
Ethionamide	Second line	Nicotinamide derivative	ethA, ethR, inhA, fabG1	(53)
