# Supplementary material for: Machine learning and phylogenetic analysis allow for predicting antibiotic resistance in M. tuberculosis
Source: BMC Microbiol. 2023 Dec 20;23:404. doi: 10.1186/s12866-023-03147-7 (PMC10731705; doi:10.1186/s12866-023-03147-7)
Supplement: Supplementary file 1 — Additional file 1. [file 12866_2023_3147_MOESM1_ESM.zip › Supplement_2.pdf]

Supplement 2

Table 1: Significantly associated mutations found with pyseer.

**Mutation**	**LRT P-value**	**Product**	**Gene**	**Start-End**
Amikacin (bonferroni corrected p-value threshold = 0.05/(no of variants) = 0.05/6551 = 7.63 x 10e-6)				
1473246, 'A,G', 'snp'(A1401G)	3.77E-91	16S ribosomal RNA	rrs	1471845-1473382
1605149, 'C,T', 'snp'(P91L)	6.94E-07	hypothetical protein	-	1604878-1606146
Capreomycin (bonferroni corrected p-value threshold = 0.05/(no of variants) = 0.05/6479 = 7.72 x 10e-6)				
1473246, 'A,G', 'snp'(A1401G)	5.35E-61	16S ribosomal RNA	rrs	1471845-1473382
Ethionamide (bonferroni corrected p-value threshold = 0.05/(no of variants) = 0.05/6821 = 7.33 x 10e-6)				
1673425, 'C,T', 'snp'(C-15T)	3.29E-11	Upstream of fabG1(1673440..1674183)	-	1673440-1674183
1473246, 'A,G', 'snp'(A1401G)	6.06E-07	16S ribosomal RNA	rrs	1471845-1473382
Kanamycin (bonferroni corrected p-value threshold = 0.05/(no of variants) = 0.05/6505 = 7.69 x 10e-6)				
1473246, 'A,G', 'snp'(A1401G)	3.30E-51	16S ribosomal RNA	rrs	1471845-1473382
2715346, 'G,A', 'snp'(C-14T)	3.99E-13	Downstream of eis(2714124..2715332)	-	2714124-2715332
2715342, 'C,T', 'snp'(G-10A)	1.02E-09	Downstream of eis(2714124..2715332)	-	2714124-2715332
Ofloxacin (bonferroni corrected p-value threshold = 0.05/(no of variants) = 0.05/7102 = 7.04 x 10e-6)				
7585, 'G,C', 'snp'(S95T)	2.02E-22	DNA gyrase subunit A	gyrA	7302-9818
7570, 'C,T', 'snp'(A90V)	2.44E-18	DNA gyrase subunit A	gyrA	7302-9818
Streptomycin (bonferroni corrected p-value threshold = 0.05/(no of variants) = 0.05/7916 = 6.32 x 10e-6)				
781687, 'A,G', 'snp'(K43R)	8.43E-47	30S ribosomal protein S12	rpsL	781560-781934
781822, 'A,G', 'snp'(K88R)	4.49E-30	30S ribosomal protein S12	rpsL	781560-781934
1472359, 'A,C', 'snp'(A514C)	1.41E-22	16S ribosomal RNA	rrs	1471845-1473382
2155168, 'C,G', 'snp'(S315T)	1.41E-15	catalase-peroxidase	katG	2153889-2156111
2719057, 'G,A', 'snp'(G-26A)	8.40E-07	Upstream of hypothetical protein	-	2719083-2719355
1673425, 'C,T', 'snp'(C-15T)	9.24E-07	Upstream of fabG1(1673440..1674183)	-	1673440-1674183
4187063, 'G,A', 'snp'(G144R)	1.97E-06	AraC/XylS family transcriptional regulator	-	4186634-4187695
1674048, 'G,A', 'snp'(L203L)	3.50E-06	3-oxoacyl-ACP reductase FabG	fabG1	1673440-1674183
