# Supplementary material for: Machine learning and phylogenetic analysis allow for predicting antibiotic resistance in M. tuberculosis
Source: BMC Microbiol. 2023 Dec 20;23:404. doi: 10.1186/s12866-023-03147-7 (PMC10731705; doi:10.1186/s12866-023-03147-7)
Supplement: Supplementary file 1 — Additional file 1. [file 12866_2023_3147_MOESM1_ESM.zip › Supplement_3.pdf]

Supplement 3

Table 1: Structural classification of known resistance-associated and novel predictive variants SVM.

Mutation	Type	Effect	Gene	Product	CARD Database	Uniprot Accession
			**Amikacin**			
1472362, 'C,T', 'snp'	rRNA	non coding transcript variant	rrs	16S ribosomal RNA	Amino-glycoside	-
			**Capreomycin**			
1472362, 'C,T', 'snp'	rRNA	non coding transcript variant	rrs	16S ribosomal RNA	Amino-glycoside	-
			**Ethionamide**			
906857, 'A,G', 'snp'	CDS	missense variant c,435A>G p,Ile145Met	pabC	4-amino-4-deoxychorismate lyase	-	Q79FW0
1472362, 'C,T', 'snp'	rRNA	non coding transcript variant	rrs	16S ribosomal RNA	Amino-glycoside	-
1673425, 'C,T', 'snp'	-	-	upstream of fabG1	-	Ethionamide	P9WGT3
			**Kanamycin**			
1472362, 'C,T', 'snp'	rRNA	non coding transcript variant	rrs	16S ribosomal RNA	Amino-glycoside	-
			**Ofloxacin**			
7570, 'C,T', 'snp'	CDS	missense variant c,269C>T p,Ala90Val	gyrA	DNA gyrase subunit A	Fluoro-quinolone	P9WG47
7362, 'G,C', 'snp'	CDS	missense variant c,61G>C p,Glu21Gln	gyrA	DNA gyrase subunit A	Fluoro-quinolone	P9WG47
			**Streptomycin**			
781395, 'T,C', 'snp'	-	-	165bp upstream of rpsL	-	Streptomycin	P9WH63

Table 1: Structural classification of known resistance-associated and novel predictive variants SVM.

2154724, 'C,A', 'snp'	CDS	missense variant c,1388G>T p,Arg463Leu	katG	catalase-peroxidase	Isoniazid & Prothionamide	P9WIE5

Table 2: Structural classification of known resistance-associated and novel predictive variants RF.

Mutation	Type	Effect	Gene	Product	CARD Database	Uniprot Accession
			**Amikacin**			
1472362, 'C,T', 'snp'	rRNA	non coding transcript variant	rrs	16S ribosomal RNA	Amino-glycoside	-
			**Capreomycin**			
1472362, 'C,T', 'snp'	rRNA	non coding transcript variant	rrs	16S ribosomal RNA	Amino-glycoside	-
			**Ethionamide**			
1670814, 'C,T', 'snp'	CDS	synonymous variant c,402C>T p,Gly134Gly	-	hypothetical protein	-	P9WLX5
1472362, 'C,T', 'snp'	rRNA	non coding transcript variant	rrs	16S ribosomal RNA	Amino-glycoside	-
			**Kanamycin**			
1472362, 'C,T', 'snp'	rRNA	non coding transcript variant	rrs	16S ribosomal RNA	Amino-glycoside	-
2714366, 'C,A', 'snp'	CDS	missense variant	eis	enhanced intracellular survival protein	Kanamycin	-
			**Ofloxacin**			
7570, 'C,T', 'snp'	CDS	missense variant c,269C>T p,Ala90Val	gyrA	DNA gyrase subunit A	Fluoro-quinolone	P9WG47
7362, 'G,C', 'snp'	CDS	missense variant c,61G>C p,Glu21Gln	gyrA	DNA gyrase subunit A	Fluoro-quinolone	P9WG47
			**Streptomycin**			
781395, 'T,C', 'snp'	-	-	165bp upstream of rpsL	-	Streptomycin	P9WH63
2154724, 'C,A', 'snp'	CDS	missense variant c,1388G>T p,Arg463Leu	katG	catalase-peroxidase	Isoniazid & Prothion-amide	P9WIE5
781687, 'A,G', 'snp'	CDS	missense variant c,128A>G p,Lys43Arg	rpsL	30S ribosomal protein S12	Streptomycin	P9WH63
1471659, 'C,T', 'snp'	ncRNA	non coding transcript variant	mcr3	Putative small regulatory RNA	-	-
