# Supplementary material for: Machine learning and phylogenetic analysis allow for predicting antibiotic resistance in M. tuberculosis
Source: BMC Microbiol. 2023 Dec 20;23:404. doi: 10.1186/s12866-023-03147-7 (PMC10731705; doi:10.1186/s12866-023-03147-7)
Supplement: Supplementary file 1 — Additional file 1. [file 12866_2023_3147_MOESM1_ESM.zip › Supplement_4.pdf]

Supplement 4

Table 1: Faulty strains that are not used for this experiment

1773.2540	1773.2720	1773.2950	1773.3400	1773.3490	1773.200	1773.2560
1773.2730	1773.2800	1773.2870	1773.2890	1773.3200	1773.3320	1773.3330
1773.3380	1773.3430	1773.3530	1773.3570	1773.3660	1773.370	1773.3770
1773.4990	1773.5000	1773.5010	1773.5020	1773.5030	1773.5040	1773.5050
1773.5070	1773.5080	1773.5090	1773.5100	1773.5120	1773.5130	1773.5140
1773.5160	1773.5170	1773.5180	1773.5190	1773.5200	1773.5210	1773.5220
1773.5240	1773.5250	1773.5260	1773.5280	1773.5290	1773.5300	1773.5310
1773.5330	1773.5340	1773.5350	1773.5360	1773.5370	1773.5380	1773.5390
1773.5410	1773.5420	1773.5430	1773.5440	1773.5450	1773.5930	

Table 2: Strains that are used for this experiment

1423431.3	1773.15623	1773.5349	1773.3381	1773.4631	1448582.3	1773.14782
1448819.3	1773.4856	1773.4066	1295753.3	1773.1493	1773.2998	1773.2742
1773.5399	1773.15074	1773.15469	1773.2946	1773.255	1773.14764	1773.2858
1427196.3	1448512.3	1773.16089	1773.2944	1447451.3	1448730.3	1447516.3
1773.14979	1773.15457	1773.15812	1773.2786	1773.15727	1773.2562	1773.2836
1773.2735	1354165.3	1773.5725	1773.5573	1400873.3	1773.2947	1447520.3
1773.15906	1773.15534	1773.15155	1773.15142	1773.15687	1773.309	1773.5205
1773.15434	1773.5596	1423534.3	1773.5607	1402589.3	1773.5465	1448655.3
1773.2682	1354175.3	1773.5342	1773.4221	1438868.3	1773.3663	1773.14867
1773.14953	1773.15098	1773.15329	1422047.3	1773.422	1773.15086	1773.3391
1773.2721	1773.15579	1773.15788	1773.15291	1773.15905	1448404.3	1773.5249
1773.5351	1773.15823	1773.3179	1448443.3	1448462.3	1324276.3	1773.5366
1448648.3	1773.3516	1773.1476	1773.16088	1773.5268	1354106.3	1773.5145
1773.2986	1773.3832	1773.15369	1773.5184	1427292.3	1773.2539	1773.3526
1773.1508	1773.5568	1773.5663	1773.2461	1773.4211	1773.15701	1773.15836
1773.343	1773.3343	1773.15931	1773.15884	1773.3339	1773.15618	1397869.3
1773.5219	1455291.3	1773.15092	1773.591	1773.15595	1324252.3	1773.16026
1773.3392	1324224.3	1773.15974	1448662.3	1773.2708	1773.15219	1773.3385
1773.15935	1773.5545	1773.2768	1773.564	1773.1475	1448822.3	1773.15322
1773.15238	1448580.3	1423504.3	1773.15279	1423427.3	1447466.3	1773.15048
1295772.3	1773.5038	1267362.3	1448652.3	1773.15689	1448680.3	1773.3347
1447474.3	1324229.3	1773.5218	1408940.4	1773.15961	1448530.3	1427206.3
1448547.3	1773.16072	1773.5165	1397886.3	1427200.3	1773.15056	1773.15965
1773.2731	1448631.3	1773.4866	1773.3314	1397923.3	1408960.4	1773.3135
1773.15902	1773.572	1773.14998	1773.5467	1773.15295	1773.5659	1773.15281
1400925.3	1773.2759	1427210.3	1773.15787	1773.2799	1773.3486	1773.5425
1773.15143	1773.3216	1773.3155	1402584.3	1397905.3	1773.14738	1773.14954
1773.15801	1773.5579	1402600.3	1402592.3	1773.3007	1773.4995	1773.4996
1397938.3	1773.15675	1773.4027	1773.16044	1773.158	1773.5649	1773.15567
1773.14935	1773.14808	1773.14962	1773.5461	1773.2664	1448588.3	1448463.3
1773.2737	1773.3336	1773.2707	1422080.3	1773.5073	1773.5259	1448596.3
1773.15413	1773.15521	1448465.3	1773.3113	1773.16087	1354102.3	1773.3475
1448565.3	1773.15437	1773.2471	1773.5681	1773.1533	1773.15793	1773.16018
1295795.3	1397913.3	1773.3358	1448427.3	1773.14947	1422040.3	1773.5561
1773.3138	1773.14916	1773.15171	1773.5043	1773.5718	1773.3042	1773.15755
1773.5035	1402583.3	1773.3459	1773.3163	1773.14754	1773.2671	1773.2781
1267357.3	1773.15706	1773.15033	1773.14876	1773.5237	1773.15769	1773.2872
1773.3227	1773.5186	1773.2969	1455283.3	1267358.3	1773.4851	1773.3268
1773.5884	1773.2848	1324219.3	1447447.3	1448610.3	1773.14889	1773.14835
1773.3164	1773.15735	1773.563	1773.4202	1267355.3	1397881.3	1408907.4
1397935.3	1773.2815	1773.5577	1773.2655	1354114.3	1773.5614	1773.5212
1773.15373	1773.15639	1773.2772	1447513.3	1448470.3	1423523.3	1402599.3

Table 2: Strains that are used for this experiment

1400898.3	1354172.3	1773.2744	1773.5002	1773.15899	1773.5441	1773.15065
1773.15976	1773.2722	1427274.3	1773.15293	1773.5123	1773.5908	1773.1516
1773.15851	652616.4	1773.15506	1773.342	1423549.3	1773.1535	1448516.3
1773.1528	1773.3462	1773.2891	1773.16075	1773.3369	1773.2938	1773.3028
1773.5174	1773.2908	1773.5554	1773.5108	1773.15023	1773.3105	1773.15568
1773.3307	1773.2616	1773.3037	1773.3186	1773.346	1448687.3	1773.589
1773.2802	1773.28	1773.15085	1423499.3	1422038.3	1773.15471	1773.332
1773.5541	1773.3025	1324243.3	1448711.3	1773.2979	1773.4644	1773.266
1448810.3	1354126.3	1773.4223	1773.5583	1773.15504	1773.15345	1421993.3
1427304.3	1773.5201	1773.5893	1773.5481	1295798.3	1773.5408	1773.2904
1773.2658	1773.5152	1427216.3	1773.506	1400935.3	1773.2514	1773.5076
1773.15201	1773.322	1773.15632	1773.14873	1773.4255	1773.15942	1448630.3
1773.2876	1773.3253	1773.1481	1427310.3	1773.2753	1295751.3	1773.5292
1773.2985	1773.5348	1773.15859	1427262.3	1773.15308	1773.14736	1773.3271
1773.1487	1773.267	1773.423	1448455.3	1773.4261	1773.5548	1773.3308
1448805.3	1773.15726	1773.16081	1773.286	1773.15784	1448417.3	1448704.3
1773.5341	1773.5175	1324270.3	1773.15165	1295763.3	1773.5046	1773.2948
1773.2959	1773.15371	1423502.3	1773.3353	1773.5322	1773.5538	1773.3177
1354111.3	1448612.3	1773.5198	1773.5615	1448722.3	1773.16102	1773.14863
1773.5293	1418256.3	1400879.3	1773.15362	1773.16096	1773.15853	1422016.3
1324271.3	1773.529	1773.15821	1773.3324	1773.3187	1397929.3	1773.1512
1773.157	1773.2835	1773.334	1397855.3	1773.5319	1448651.3	1773.4616
1773.15739	1400884.3	1773.16019	1773.14922	1773.3383	1773.1595	1773.537
1773.3348	1773.5295	1773.15709	1324290.3	1773.15712	1773.3356	1773.15594
1773.3404	1773.15745	1773.3685	1354127.3	1773.3313	1400909.3	1773.14739
1354135.3	1773.15359	1773.306	1773.4164	1773.16048	1773.2639	1773.15138
1773.296	1773.15344	1773.15224	1773.317	1773.2559	1773.2967	1773.5122
1773.16028	1773.2907	1422084.3	1295740.3	1447501.3	1427330.3	1773.5515
1773.15062	1773.2436	1773.15667	1773.519	1773.15686	1448633.3	1773.5118
1423514.3	1773.15981	1354137.3	1773.2494	1423510.3	1773.2608	1448634.3
1773.3597	1773.1537	1427261.3	1773.5163	1448783.3	1773.15167	1773.15338
1773.15862	1773.14796	1773.3512	1427325.3	1773.275	1773.546	1427322.3
1408932.4	33894.5	1773.15406	1448394.3	1773.16002	1773.3421	1773.16043
1448697.3	1773.347	1773.2521	1773.4219	1773.1536	1773.14855	1773.3092
1423506.3	1423539.3	1773.499	1447449.3	1447488.3	1773.14886	1773.5331
1354132.3	1773.334	1423425.3	1773.5095	1448738.3	1773.2893	1773.3087
1773.2554	1422100.3	1773.15251	1773.2874	1773.3053	1773.515	1773.14853
1773.5507	1773.3346	1773.4855	1773.2612	1773.15585	1397863.3	1773.3444
1773.5458	1773.4208	1773.3522	1408974.4	1423520.3	1773.2489	1773.5139
1773.4321	1773.5657	1422029.3	1773.3282	1773.15464	1773.289	1448617.3
1773.2706	1400870.3	1773.2439	1773.5469	1773.3056	1773.5707	1773.15218
1448645.3	1438837.3	1438839.3	1324235.3	1773.5624	1773.1486	1773.2649

Table 2: Strains that are used for this experiment

1773.15877	1397861.3	1448597.3	1773.15263	1448641.3	1773.15375	1773.5365
1448732.3	1773.15454	1773.2541	1773.2469	1773.5921	1324225.3	1773.1573
1448795.3	1448694.3	1773.2523	1773.4989	1773.3491	1448791.3	1773.5771
1773.328	1773.2971	1773.5414	1773.15777	1773.3172	1773.5453	1773.2784
1448627.3	1773.15144	1448787.3	1773.3036	1773.324	1448757.3	1773.14818
1773.15229	1773.4535	1773.5162	1447509.3	1773.15031	1773.15614	1773.3088
1773.15447	1773.333	1773.15636	1773.14903	1773.3406	1773.15805	1408936.4
1773.15284	1773.15306	1773.16013	1408965.4	1448827.3	1773.14806	1773.5379
1773.2779	1447496.3	1400933.3	1295792.3	1427252.3	1773.15876	1773.15882
1773.245	1773.5906	1773.2796	1773.2582	1773.16016	1773.3015	1427281.3
1448473.3	1773.3267	1773.5387	1773.5261	1448399.3	1354101.3	1448671.3
1773.15937	1295726.3	1773.15729	1773.2983	1773.5604	1773.3396	1773.15005
1773.5179	1773.5555	1773.2468	1448778.3	1448430.3	1773.5364	1773.3467
1773.15828	1438849.3	1773.2879	1773.14931	1447435.3	1773.5109	1773.14958
1773.16074	1448406.3	1773.5407	1397860.3	1773.5224	1773.2776	1773.3182
1773.3153	1427319.3	1773.15184	1773.15079	1773.501	1448586.3	1773.14978
1773.2622	1773.3259	1773.1494	1773.14951	1773.5182	1773.14898	1773.2524
1324264.3	1773.326	1773.15282	1417011.3	1773.338	1773.2766	1773.4248
1448567.3	1447436.3	1773.3694	1773.15888	1773.3258	1773.15334	1773.3364
1438863.3	1773.15517	1408937.4	1773.3074	1773.5029	1773.2578	1773.3408
1773.5719	1773.15361	1427312.3	1417018.3	1400940.3	1438843.3	1448640.3
1400930.3	1354155.3	1773.503	1773.3454	1354120.3	1773.3114	1773.3251
1400871.3	1773.3357	1773.5333	1773.4228	1773.15772	1773.59	1773.3151
1773.2634	1773.2989	1773.363	1773.43	1773.15479	1448632.3	1773.15298
1773.5521	1448434.3	1773.5433	1773.2982	1773.15059	1773.5164	1773.3688
1773.2853	1773.14974	1773.4667	1773.5083	1773.5241	1773.16038	1773.5648
1773.296	1400886.3	1773.15699	1773.5383	1773.5508	1427307.3	1773.1491
1773.4658	1773.15159	1773.2643	1773.14755	1773.3333	1448491.3	1773.15057
1773.3703	1773.15814	1773.2691	1773.329	1773.5647	1417009.3	1448821.3
1267360.3	1422079.3	1455266.3	1397901.3	1773.5567	1397897.3	1773.3249
1427209.3	1773.14983	1773.5172	1427250.3	1773.15575	1773.2566	1773.2673
1408927.4	1773.15909	1773.2618	1427207.3	1773.15496	1423554.3	1773.5033
1400874.3	1773.5042	1773.15197	1773.15307	1773.16069	1773.16077	1773.2477
1773.31	1773.4031	1438881.3	1773.3418	1455263.3	1773.15102	1773.199
1773.312	1773.3402	1438842.3	1773.15529	1773.15679	1354116.3	1773.5302
1773.2683	1773.5142	1421997.3	1773.2736	1354107.3	1773.4236	1400921.3
1773.14915	1773.4241	1773.2647	1455278.3	1324277.3	1773.333	1773.5602
1773.2995	1773.2486	1773.14893	1773.2642	1423516.3	1773.3184	1773.3201
1773.3012	1773.5084	1773.2987	1423428.3	1773.15409	1773.15682	1408918.4
1400924.3	1773.2728	1773.5251	1773.15638	1773.5313	1773.15356	1773.5041
1448807.3	1773.2687	1773.14762	1773.34	1773.2452	1773.15954	1773.285
1773.15725	1773.5713	1421966.3	1773.486	1773.3142	1773.3171	1773.4671

Table 2: Strains that are used for this experiment

1427221.3	1773.5473	1427286.3	1773.15052	1773.2764	1773.2831	1773.15016
1773.2747	1773.14913	1773.5459	1773.5697	1773.15527	1773.3102	1773.2718
1455311.3	1773.3131	1773.15025	1773.15874	1773.15047	1447444.3	1422069.3
1773.15841	1773.2725	1773.3513	1408967.4	1773.516	1773.5355	1773.151
1773.5885	1773.5239	1455308.3	1773.14929	1773.15207	1773.5101	1773.14791
1402595.3	1773.4598	1773.251	1773.3132	1773.2481	1773.3805	1773.15538
1773.15401	1773.2656	1773.5724	1773.3432	1324228.3	1773.5194	1773.15995
1773.14793	1354119.3	1773.3523	1773.5024	1427219.3	1773.2873	1417029.3
1773.5196	1773.15939	1773.557	1773.5327	1773.2446	1773.3683	1773.15957
1773.3248	1773.5317	1773.3424	1448690.3	1773.5661	1773.14923	1455292.3
1422098.3	1773.2677	1447465.3	1773.5204	1773.15254	1427257.3	1773.15318
1773.3305	1773.3162	1448439.3	1773.2637	1773.3083	1773.3754	1354123.3
1397878.3	1448615.3	1773.15449	1773.244	1448428.3	1773.15809	1773.2662
1773.15549	1773.3269	1773.5535	1773.15768	1773.4225	1773.2589	1408963.4
1423573.3	1448743.3	1773.15163	1397880.3	1773.1507	1773.15843	1773.4676
1773.2571	1773.15834	1773.15	1773.331	1773.2698	1773.5501	1773.14992
1773.15536	1773.2757	1324232.3	1448616.3	1448643.3	1773.2867	1448623.3
1773.4026	1773.2743	1773.2719	1448480.3	1773.538	1773.343	1773.5493
1773.15332	1773.32	1408928.4	1773.4099	1773.3206	1773.2638	1773.5622
1427249.3	1773.15578	1773.15858	1773.1506	1427227.3	1448663.3	1773.5087
1423483.3	1773.5621	1324250.3	1295747.3	1773.16061	1773.15475	1773.5353
1773.26	1773.2762	1773.2838	1397890.3	1448808.3	1448482.3	1773.15278
1773.16032	1773.1498	1448577.3	1773.15598	1773.2565	1773.34	1421937.3
1295721.3	1773.14819	1408946.4	1773.15091	1773.15237	1773.15685	1773.2792
1438865.3	1448744.3	1773.3502	1773.15762	1773.3051	1447457.3	1324288.3
1773.3442	1427289.3	1773.16036	1295782.3	1427246.3	1773.15582	1773.5345
1773.15695	1773.15456	1773.276	1773.1565	1773.5308	1773.15017	1773.3458
1423451.3	1773.5055	1773.16	1773.15615	1773.15069	1773.1532	1773.3062
1773.3411	1773.5323	1773.5682	1773.3104	1773.15034	1773.3974	1773.2845
1773.2604	1773.5729	1773.15616	1773.14996	1773.5901	1448800.3	1773.2599
1773.3024	1773.15134	1423437.3	1773.5914	1773.5173	1448431.3	1773.3241
1773.15013	1773.14804	1773.14817	1773.281	1408916.4	1773.3011	1773.408
1773.15335	1773.204	1773.2507	1773.15014	1773.5114	1447434.3	1773.4994
1773.5311	1423541.3	1773.15001	1773.15273	1397941.3	1773.1477	1773.2928
1448620.3	1773.535	1773.15393	1773.14881	1773.15407	1773.3158	1773.16066
1773.2923	1448529.3	1773.5257	1773.5601	1773.2528	1773.15436	1295756.3
1773.2579	1397909.3	1773.3449	1773.15794	1773.31	1773.5575	1773.3303
1773.15977	1773.293	1397916.3	1773.5557	1427235.3	1408924.4	1773.3359
1773.5085	1773.14899	1773.15721	1773.2789	1447476.3	1773.3335	1324260.3
1427189.3	1448678.3	1773.15495	1773.15389	1423481.3	1773.15959	1773.15766
1773.14949	1773.15055	1773.3316	1354145.3	1417010.3	1408955.4	1402590.3
1773.16054	1773.3471	1448668.3	1773.1521	1773.4246	1773.5675	1427323.3

Table 2: Strains that are used for this experiment

1773.3372	1773.5384	1400937.3	1448669.3	1773.3243	1773.15039	1773.5484
1422012.3	1773.15924	1773.15374	1773.3289	1324275.3	1354191.3	1773.5026
1773.15952	1773.5017	1773.2503	1324237.3	1773.351	1448532.3	1295799.3
1773.534	1448407.3	1422036.3	1427268.3	1354143.3	1773.5032	1422021.3
1773.1578	1773.5576	1448672.3	1773.3079	1773.4209	1773.4865	1773.5556
1773.2459	1773.2964	1324281.3	1773.2869	1773.1583	1773.4992	1773.5479
1773.5421	1427202.3	1773.2686	1447504.3	1773.15182	1773.3531	1773.2517
1448595.3	1773.14742	1773.15663	1773.313	1773.15603	1773.3439	1773.3119
1773.3254	1427236.3	1773.5539	1421941.3	1773.249	1773.3436	1773.5111
1773.15149	1354105.3	1773.3069	1773.15833	1773.1545	1773.359	1773.3043
1354156.3	1423461.3	1773.16071	1354162.3	1448673.3	1773.14956	1773.15722
1773.5405	1423438.3	1427315.3	1773.5022	1773.349	1773.523	1773.15708
1397910.3	1324234.4	1773.14975	1773.3334	1773.5668	1773.14779	1773.15605
1324220.3	1421988.3	1422093.3	1438871.3	1448642.3	1408964.4	1773.5018
1438869.3	1402597.3	1324300.3	1773.14973	1773.15566	1773.15928	1773.3484
1397853.3	1773.5432	1324242.3	1427224.3	1773.5171	1324253.3	1773.5234
1773.327	1773.2601	1773.15106	1773.15491	1773.15478	1773.15494	1773.15811
1773.5157	1773.2652	1773.15419	1408970.4	1773.15114	1421996.3	1773.5028
1773.525	1448752.3	1773.2714	1438833.3	1773.15966	1773.1594	1427187.3
1773.3696	1773.4862	1773.5392	1773.3678	1773.5316	1773.2657	1773.1543
1773.3337	1773.3166	1773.2788	1448784.3	1773.2949	1773.15511	1397877.3
1773.15804	1773.15872	1773.2861	1773.5662	1397900.3	1773.4148	1423536.3
1773.15412	1354187.3	1773.3503	1773.256	1773.15629	1773.5066	1447502.3
1773.3077	1773.14926	1773.1538	1773.4104	1773.1534	1773.15822	1773.15038
1773.3524	1773.15022	1448734.3	1773.5471	1773.3045	1422061.3	1773.1599
1773.15938	1773.355	1455285.3	1448695.3	1397926.3	1448709.3	1773.16034
1773.3447	1773.1577	1773.15271	1773.15141	1773.2457	1773.2822	1448799.3
1773.14985	1773.1589	1773.325	1427278.3	1773.2775	1773.3021	1773.53
1773.5426	1773.15315	1773.14937	1773.5135	1447459.3	1773.2826	1448790.3
1400923.3	1397933.3	1423535.3	1423484.3	1773.15379	1773.3199	1448755.3
1773.2513	1448562.3	1773.15657	1773.15045	1773.15168	1773.2999	1447485.3
1773.14984	1773.5089	1773.4229	1773.253	1448600.3	1427179.3	1423530.3
1397893.3	1773.15274	1397924.3	1773.2827	1773.15577	1773.14737	1773.5256
1773.16004	1773.5529	1773.3493	1455293.3	1354153.3	1773.15199	1773.2623
1773.5448	1773.15357	1773.3157	1773.16029	1773.1479	1773.14829	1448552.3
1448637.7	1773.593	1773.2635	1773.2996	1773.3485	1773.2881	1773.15265
1773.3147	1773.5007	1448592.3	1773.2791	1773.4845	1773.5312	1773.15664
1773.2555	1397934.3	1773.15312	1773.15123	1773.14797	1773.323	1773.5635
1773.15223	1773.3223	1773.2479	1773.308	1773.2614	1773.5008	1448660.3
1773.14758	1773.518	1773.3103	1427225.3	1773.15641	1423555.3	1773.1567
1400936.3	1397872.3	1773.275	1773.15723	1773.14977	1773.5702	1773.4691
1773.2512	1773.5283	1773.4858	1773.2505	1773.3287	1773.16094	1773.315

Table 2: Strains that are used for this experiment

1773.2941	1773.15544	1773.3455	1773.1605	1773.15376	1397857.3	1295801.3
1402601.3	1773.3302	1773.5487	1773.5148	1448740.3	1438860.3	1773.15651
1773.15994	1354152.3	1773.15126	1773.15767	1773.4207	1773.1557	1773.2777
1427314.3	1773.357	1773.5126	1773.5156	1773.5913	1773.5206	1773.5564
1773.5715	1423432.3	1354121.3	1773.3244	1773.14787	1773.4049	1773.3174
1773.14816	1773.15445	1773.2485	1773.532	1295803.3	1773.15466	1773.284
1773.5009	1773.2825	1773.2897	1773.5887	1773.14775	1408909.4	1773.16053
1773.15314	1438854.3	1422034.3	1773.15222	1773.3274	1773.3415	1773.5039
1773.14909	1773.15531	1448585.3	1773.14823	1773.14842	1773.15856	1773.15267
1448572.3	1448479.3	1354174.3	1773.2899	1773.5571	1773.5199	1397882.3
1773.15264	1773.5131	1397895.3	1773.15619	1773.3169	1773.2733	1354142.3
1773.15428	1422001.3	1773.14892	1773.14788	1447521.3	1773.2627	1773.14765
1773.2669	1773.5488	1773.15826	1773.3231	1773.15127	1773.2894	1438840.3
1773.15532	1773.15396	1400920.3	1773.15489	1324282.3	1773.14884	1773.5346
1448727.3	1773.15848	1448446.3	1773.2467	1773.2843	1427256.3	1421956.3
1773.377	1448703.3	1773.2833	1773.15626	1448767.3	1773.15728	1773.5422
1773.15989	1773.15019	1773.16091	1773.2864	1773.14843	1427272.3	1447452.3
1447454.3	1773.2535	1773.5047	1773.15512	1773.2586	1773.2756	1773.5578
1397915.3	1773.5656	1773.15969	1773.14826	1773.16006	1773.4991	1448656.3
1773.3136	1447493.3	1773.556	1427313.3	1773.1581	1397889.3	1448590.3
1773.292	1773.5644	1773.15658	1773.1513	1773.2727	1773.325	1773.3544
1354147.3	1773.15081	1773.5613	1400931.3	1773.14838	1773.5093	1324254.3
1773.15798	1773.3351	1773.14813	1455284.3	1423547.3	1448624.3	1773.4184
1773.2745	1447503.3	1421977.3	1448771.3	1267356.3	1773.2854	1773.15026
1773.2975	1423572.3	1773.14802	1773.2771	1773.15714	1448716.3	1773.5258
1773.15786	1448676.3	1773.313	1773.15569	1773.15733	1773.15443	1422022.3
1408954.4	1773.2787	1397864.3	1773.4214	1773.4252	1773.3483	1773.1553
1400929.3	1773.4993	1773.5217	1773.15718	1773.15508	1773.14894	1448537.3
1408923.4	1438832.3	1773.3842	1773.15277	1773.265	1773.1566	1448400.3
1402596.3	1400938.3	1773.2818	1773.15209	1773.3414	1324255.3	1773.5226
1773.36	1773.5606	1773.2451	1773.5496	1773.5284	1448691.3	1773.14921
1417027.3	1773.15964	1324263.3	1448628.3	1773.4201	1448677.3	1773.514
1773.15477	1773.261	1773.14732	1773.2924	1773.15458	1448598.3	1423430.3
1773.337	1427329.3	1408943.4	1773.14885	1773.5666	1773.5291	1427203.3
1773.14801	1448509.3	1773.2935	1773.14887	1773.1572	1773.3121	1773.2619
1423542.3	1438867.3	1773.3246	1773.5192	1423519.3	1773.2763	1427230.3
1773.3255	1447489.3	1773.4707	1773.5034	1448397.3	1773.14828	1773.559
1773.349	1397918.3	1773.15455	1427290.3	1773.3413	1773.15011	1773.14777
1773.524	1773.5406	1773.16039	1773.4247	1773.5611	1438851.3	1773.3228
1773.4756	1324293.3	1773.15704	1773.536	1773.1569	1773.3328	1773.15707
1448688.3	1773.15135	1773.5486	1423521.3	1448753.3	1422042.3	1773.5252
1773.3451	1773.3378	1773.14868	1773.1579	1773.338	1773.3435	1773.4836

Table 2: Strains that are used for this experiment

1773.2552	1402588.3	1354181.3	1427193.3	1773.15655	1455275.3	1773.5098
1324231.3	1773.14875	1773.15094	1773.15925	1773.15975	1773.15986	1448806.3
1448604.3	1773.14933	1773.3237	1773.15252	1773.3474	1773.5338	1448548.3
1397868.3	1773.4998	1773.15333	1773.27	1773.5706	1773.3175	1295727.3
1773.15581	1773.5625	1773.2515	1773.5594	1773.5504	1773.5442	1773.5549
1773.3048	1773.4861	1324284.3	1773.15813	1773.15863	1773.15444	1773.14784
1773.5413	1448720.3	1773.15051	1773.2939	1773.504	1773.15364	1773.14776
1773.522	1773.15107	1448707.3	1773.15551	1773.2816	1773.356	1773.3176
1773.15742	1773.15174	1773.15599	1773.2993	1295744.3	1417016.3	1773.3115
1400891.3	1773.15442	1773.15831	1397856.3	1773.5376	1773.5686	1773.554
1448405.3	1448555.3	1773.15286	1448769.3	1773.3245	1427181.3	1773.1542
1773.15525	1773.15351	1773.3332	1448587.3	1773.15557	1773.5069	1773.3478
1773.3001	1773.15462	1773.14845	1773.326	1773.15082	1773.15485	1773.15871
1773.15122	1773.15771	1773.2895	1773.3509	1773.5723	1773.3398	1773.301
1773.336	1773.5455	1773.3533	1773.3393	1418255.3	1773.14807	1400880.3
1773.3211	1773.15941	1773.16086	1773.5558	1773.3204	1773.15006	1773.2499
1773.5572	1773.15275	1773.4212	1773.4206	1773.3055	1773.2448	1773.5207
1773.3202	1773.15121	1773.15962	1773.4234	1773.15463	1773.15151	1773.294
1773.15347	1773.3529	1448527.3	1773.2475	1773.15988	1773.3029	1422052.3
1773.5103	1773.2807	1773.15272	1773.3107	1773.3152	1773.5144	1773.2887
1773.15024	1773.16058	1448715.3	1773.15063	1773.14989	1773.15608	1773.2564
1773.3527	1773.15246	1773.2463	1773.5597	1773.5282	1773.1555	1773.15073
1773.16025	1773.3183	1773.16008	1427180.3	1773.3165	1773.1525	1773.1576
1448705.3	1773.15546	1773.15021	1448609.3	1773.15593	1773.3133	1773.4999
1423575.3	1448440.3	1773.15847	1448442.3	1773.15883	1773.307	1324236.3
1427270.3	1773.16031	1773.4222	1773.283	1773.2972	1773.15734	1773.15765
1773.14934	1448478.3	1773.15429	1773.2778	1773.5143	1773.15761	1773.2668
1402598.3	1773.15297	1773.2473	1773.2889	1773.3006	1397854.3	1773.2694
1773.2568	1773.5658	1773.16033	1773.263	1773.3355	1773.15241	1773.5685
1400908.3	1773.4249	1448814.3	1448498.3	1773.15423	1773.2525	1438892.3
1324245.3	1773.5616	1773.5099	1773.15673	1447461.3	1324285.3	1448584.3
1438852.3	1773.14769	1773.15388	1773.3109	1773.14849	1773.15179	1773.1519
1773.1596	1773.2834	1773.3622	1773.3437	1773.5927	1397931.3	1773.15244
1400883.3	1400934.4	1773.2509	1773.2878	1773.15932	1773.14756	1773.318
1773.553	1773.1575	1773.5298	1773.15096	1427275.3	1773.3431	1773.15648
1773.5141	1773.15173	1773.5385	1773.15919	1773.3376	1773.2703	1773.15242
1773.5159	1773.258	1773.4674	1773.5513	1448649.3	1773.15555	1448393.3
1773.2621	1402587.3	1773.14938	1773.15519	1773.15691	1773.4253	1773.3076
1773.505	1324261.3	1773.14869	1773.5514	1773.2699	1448401.3	1448725.3
1773.15795	1773.2769	1448484.3	1773.14805	1773.5589	1773.1511	1455270.3
1773.5328	1773.1518	1773.15881	1773.2533	1427273.3	1773.2527	1448461.3
1773.16045	1773.3061	1773.3097	1773.15088	1773.5079	1773.3125	1773.5003

Table 2: Strains that are used for this experiment

1773.372	1773.3433	1773.5133	1773.2774	1423570.3	1448488.3	1773.5475
1447514.3	1773.2641	1400882.3	1773.5116	1448654.3	33894.6	1773.3035
1773.2957	1447464.3	1773.14837	1773.14932	1773.364	1408941.4	1773.2491
1773.305	1422026.3	1773.5104	1397927.3	1448798.3	1773.5025	1408947.4
1773.15394	1773.3505	1773.4121	1773.15533	1773.5883	1773.2884	1423485.3
1448576.3	1773.303	1773.5178	1773.2661	1427316.3	1773.3497	1773.14945
1773.15213	1773.15328	1773.15611	1773.15835	1773.16095	1773.5464	1773.5452
1773.567	1773.3409	1400916.3	1427264.3	1773.5246	1400918.3	1773.3397
1773.2455	1773.14861	1773.2628	1773.3264	1773.5626	1773.16097	1773.5451
1773.14751	1455289.3	1773.371	1773.15634	1773.15873	1773.5652	1773.14936
1423467.3	1448789.3	1397906.3	1773.304	1773.2	1773.5014	1773.15007
1448607.3	1773.15896	1773.274	1773.15606	1773.3472	1773.1509	1773.1587
1773.5019	1773.14792	1773.15724	1773.15901	1773.421	1447471.3	1773.307
1773.15418	1448554.3	1773.5128	1773.37	1773.332	1773.15198	1773.2902
1408957.4	1773.15154	1773.2795	1773.3419	1773.277	1295790.3	1773.5263
1408968.4	1447456.3	1773.14941	1773.15903	1427228.3	1773.14744	1354173.3
1408920.4	1447460.3	1773.5266	1773.15528	1448608.3	1773.15945	1773.2752
1427229.3	1773.3375	1773.5352	1400927.3	1773.5632	1773.319	1773.526
1773.15145	1773.15576	1773.15377	1448675.3	1773.5726	1402586.3	1773.2567
1773.282	1773.15933	1423529.3	1773.15119	1773.4215	1773.15319	1773.15384
1408952.4	1773.5286	1773.15343	1773.3394	1773.14844	1773.5944	1295776.3
1773.3379	1773.3193	1773.15999	1438877.3	1427282.3	1773.15066	1773.552
1408930.4	1773.558	1423566.3	1397917.3	1448569.3	1773.15276	1773.15991
1773.3463	1773.3101	1773.5332	1773.15382	1773.1517	1773.15421	1773.14946
1408956.4	1773.2441	1773.2749	1400922.3	1408921.4	1773.1515	1773.3511
1773.5544	1773.14731	1773.15446	1773.1556	1773.2498	1773.3371	1427303.3
1773.2973	1427233.3	1773.5891	1448726.3	1448717.3	1448579.3	1773.15665
1773.468	1773.15522	1773.3126	1773.14866	1773.3331	1773.5532	1448829.3
1408934.4	1773.16057	1427251.3	1773.14907	1773.15294	1295771.3	1773.15857
1773.2968	1773.2901	1773.5138	1324294.3	1773.5498	1773.15592	1773.15719
1773.5694	1773.2444	1438873.3	1773.4038	1773.5344	1773.5297	1773.339
1448490.3	1773.2866	1448599.3	1448733.3	1773.2981	1773.15311	1438878.3
1773.3301	1448492.3	1447448.3	1448718.3	1773.1504	1773.5151	1773.15631
1773.302	1773.2716	1397859.3	1773.279	1447518.3	1773.5106	1773.15392
1773.3217	1448563.3	1773.15133	1773.3033	1354158.3	1773.2442	1773.15305
1422072.3	1397902.3	1455286.3	1773.5672	1773.2496	1773.269	1773.2758
1324274.3	1773.5362	1773.15289	1773.15124	1448546.3	1400888.3	1773.14959
1773.2767	1455272.3	1773.5185	1773.15693	1427215.3	1448647.3	1427243.3
1773.15753	1773.2773	1773.5456	1773.15979	1773.1558	1773.15431	1773.5121
1438859.3	1448817.3	1773.15221	1773.4233	1773.4864	1773.5449	1773.3341
1773.14927	1773.16035	1773.2711	1773.291	1324299.3	1773.42	1773.15927
1417017.3	1773.5339	1773.2868	1455288.3	1773.15131	1773.2898	1427213.3

Table 2: Strains that are used for this experiment

1773.5536	1773.4181	1773.5325	1448464.3	78331.98	1408962.4	1773.5651
1773.2591	1773.3367	1773.15526	1773.3004	1773.14759	1773.2915	1438879.3
1773.2859	1773.3208	1773.3521	1773.5052	1773.15824	1773.15584	1773.14948
1773.5377	1773.2574	1773.15816	1773.14912	1773.2663	1773.29	1773.5373
1773.271	1448635.3	1773.15773	1773.268	1773.15791	1773.5709	1773.3525
1295766.3	1421921.3	1324279.3	1773.14821	1773.15674	1400887.3	1773.2636
1773.3148	1773.341	1773.5419	1773.4262	1354182.3	1773.5506	1773.3468
1423503.3	1773.15752	1773.3366	1397914.3	1773.5375	1773.15507	1773.2715
1773.3515	1354130.3	1427302.3	1773.15111	1421954.3	1773.52	1773.15622
1773.15381	1773.15627	1773.15358	1773.315	1773.15146	1773.16022	1773.3338
1773.15467	1773.15003	1773.15105	1773.2794	1773.5463	1773.15089	1773.15501
1773.3466	1773.14749	1773.2502	1773.3059	1427308.3	1773.15702	1773.1526
1773.3306	1773.16068	1408906.4	1421986.3	1773.155	1423512.3	1448517.3
1773.5677	1421928.3	1773.5361	1773.14795	1408915.4	1354129.3	1354179.3
1422008.3	1773.5228	1773.4852	1422087.3	1773.2549	1447443.3	1427199.3
1448702.3	1773.15875	1422065.3	1448679.3	1427226.3	1773.548	1773.3349
1773.2583	1773.3161	1773.5637	1773.15921	1447480.3	1773.15018	1773.1478
1400881.3	1773.3377	1773.2695	1773.3943	1773.5525	1773.14815	1773.15395
1773.2732	1295745.3	1773.15352	1773.2954	1773.342	1773.4205	1773.5468
1773.15353	1773.1544	1773.3429	1354164.3	1773.3252	1773.15571	1773.2921
1773.5916	1773.3038	1773.15493	1448411.3	1773.2994	1773.16014	1773.5154
1455295.3	1773.15676	1773.3075	1773.2814	1773.285	1773.2813	1773.5693
1773.3464	1773.15996	1455264.3	1773.14785	1773.2445	1773.14761	1773.2688
1324298.3	1448453.3	1773.15189	1427305.3	1773.15637	1324265.3	1773.2817
1773.345	1773.3496	1773.5537	1773.5664	1400926.3	1773.5369	1773.3247
1773.4867	1422013.3	1448780.3	1773.15181	1448729.3	1773.1502	1400904.3
1773.2551	1773.14733	1773.5512	1773.15157	1448458.3	1773.4226	1773.1548
1773.5048	1773.5137	1447463.3	1427311.3	1773.5509	1295739.3	1773.3438
1773.1591	1773.1598	1773.3145	1773.4227	1773.5012	1773.3234	1427205.3
1354122.3	1773.3283	1773.1522	1773.14847	1773.1549	1773.2522	1773.3299
1773.15645	1773.15741	1448625.3	1295759.3	1773.14895	1773.3156	1418252.3
1397928.3	1448412.3	1448698.3	1773.5023	1773.14757	1773.15509	1397884.3
1423436.3	1423558.3	1773.14789	1773.351	1408942.4	1773.3207	1448636.3
1400939.3	1448568.3	1773.2696	1773.3461	1400872.3	1423508.3	1324295.3
1448650.3	1773.551	1397858.3	1773.3226	1773.5412	1421992.3	1455290.3
1773.3276	1408911.4	1448606.3	1354136.3	1354133.3	1773.15002	1427188.3
1448459.3	1773.5427	1773.3318	1773.4612	1773.4857	1773.5233	1448559.3
1773.5728	1773.3727	1773.3123	1773.3488	1773.314	1295796.3	1773.15552
1773.5167	1397867.3	1773.3412	1773.15192	1773.2483	1773.2958	1773.3064
1773.5495	1773.14877	1773.14746	1773.14735	1773.5247	1773.14767	1448764.3
1773.4697	1773.15336	1773.15661	1773.5524	1773.14991	1773.5683	1773.16037
1773.14888	1773.3498	1448812.3	1773.15214	1773.15607	1773.3041	1773.5215

Table 2: Strains that are used for this experiment

1773.4235	1773.5587	1448745.3	1773.16065	1773.3178	1773.14803	1421973.3
1773.15545	1773.5294	1773.5326	1773.3122	1400895.3	1773.5642	1423517.3
1773.2558	1773.521	1773.15758	1773.304	1773.3637	1773.2581	1773.3476
1423440.3	1773.15749	1448421.3	1773.2674	1773.3086	1773.15898	1773.15476
1773.3073	1773.5445	1448746.3	1773.15372	1773.5213	1773.3368	1773.15516
1408905.4	1773.5013	1354110.3	1773.2506	1773.16076	1295767.3	1773.4627
1773.3054	1773.2821	1773.15186	1773.3057	1354139.3	1773.2824	1773.3297
1773.5542	1427320.3	1773.278	1773.15194	1773.2543	1773.161	1773.2754
1773.1604	1773.15617	1773.5617	1773.2798	1773.2739	1773.5522	1773.14957
1773.5232	1773.3487	1773.3072	1408929.4	1773.5015	1773.5472	1773.15247
1773.15642	1773.3469	1773.254	1773.5574	1773.4092	1447486.3	1448796.3
1773.3869	1773.15128	1773.15897	1448402.3	1773.2672	1773.15666	1773.3489
1773.15732	1773.2548	1408922.4	1448468.3	1773.5335	1324248.3	1773.15907
1773.14904	1773.15731	1773.3386	1773.2684	1427183.3	1773.5667	1773.14741
1354109.3	1773.346	1773.15042	1773.15915	1773.5416	1773.2851	1773.4678
1397930.3	1773.15796	1423531.3	1773.2484	1773.4601	1448433.3	1448683.3
1773.15368	1773.2606	1773.14812	1773.367	1447525.3	1773.15097	1773.15497
1773.15698	1773.2462	1773.15385	1773.322	1773.3576	1773.2685	1773.3518
1773.14943	1455294.3	1773.3233	1448398.3	1773.5147	1773.5092	1773.54
1773.2631	1773.5388	1448824.3	1773.15647	1354144.3	1448639.3	1423446.3
1773.2653	1773.4844	1773.2611	1773.2885	1773.3205	1773.15228	1773.5287
1773.15523	1773.3124	1773.513	1773.5699	1421983.3	1773.2952	1324269.3
1773.14999	1773.15963	1773.2569	1324267.3	1773.5183	1397919.3	1773.5078
1773.3704	1448766.3	1773.15825	1354176.3	1773.274	1773.3284	1773.15459
1448681.3	1773.16024	1773.2723	1324233.3	1448611.3	1448524.3	1773.15785
1773.335	1773.4709	1421969.3	1448467.3	1773.2803	1448728.3	1773.3501
1773.5584	1773.3598	1773.292	1773.15139	1773.5363	1773.2797	1773.3708
1423515.3	1773.247	1773.15046	1397939.3	1773.2595	1773.14914	1447506.3
1773.5551	1773.5592	1427223.3	1773.16009	1773.5096	1422076.3	1773.16005
1773.2809	1773.3345	1773.2828	1773.2596	1427222.3	1773.15422	1773.2474
1773.5888	1773.4232	1773.15895	1773.2584	1773.5202	1773.5679	1773.4734
1397899.3	1447482.3	1427299.3	1773.5669	1773.3519	1773.15861	1773.5497
1773.2454	1773.15885	1773.16046	1773.5382	1773.1593	1448823.3	1773.5717
1773.15468	1448477.3	1773.5428	1773.3315	1773.3197	1448659.3	1438836.3
1773.5168	1427301.3	1773.15404	1773.269	1773.5037	1773.4263	1773.3013
1773.3235	1773.15913	1773.15071	1773.15101	1427214.3	1773.15482	1773.5517
1773.15004	1773.16103	1773.15044	1773.15515	1773.5907	1427280.3	1773.3751
1773.5489	1773.357	1773.5704	1422068.3	1773.5153	1324230.3	1773.3387
1773.2842	1773.5477	1448721.3	1773.3499	1773.2943	1448436.3	1773.3279
1773.4846	1773.3363	1773.3321	1354124.3	1448777.3	1773.5368	1773.15415
1773.2464	1773.2932	1773.15838	1448571.3	1773.3401	1354166.3	1295742.3
1773.15043	1423545.3	1448773.3	1448395.3	1354128.3	1773.3749	1773.5705

Table 2: Strains that are used for this experiment

1773.5136	1773.5665	1448564.3	1773.4259	1448535.3	1427317.3	1448759.3
1773.2516	1448665.3	1397873.3	1267363.3	1773.5334	1773.1529	1773.15303
1773.5527	1773.509	1773.15316	1448506.3	1773.3422	1773.15563	1773.3118
1773.3198	1773.3222	1773.2912	1448714.3	1448748.3	1448664.3	1448435.3
1773.14824	1773.15997	1448736.3	1773.3108	1773.3257	1773.3423	1427276.3
1773.14774	1448828.3	1773.545	1448751.3	1773.16007	1773.3	1773.3134
1773.15653	1773.2819	1773.3309	1773.3098	1773.15524	1773.16084	1427237.3
1773.5091	1447437.3	1773.15296	1773.2607	1773.15041	1773.5071	1773.2804
1773.5021	1773.3298	1354141.3	1773.4997	1438834.3	1773.549	1773.5582
1773.3407	1423435.3	1773.15078	1773.15649	1773.14753	1773.2726	1773.14839
1773.15232	1773.15367	1773.1523	1455307.3	1773.5639	1773.15514	1773.2447
1773.2625	1773.3262	1773.5462	1427260.3	1423562.3	1773.3291	1773.5336
1773.2748	1447508.3	1773.2903	1773.3747	1773.5599	1773.5716	1773.15596
1773.4231	1773.15317	1773.2755	1773.2919	1773.2679	1417030.3	1427288.3
1773.3159	1773.344	1773.15565	1773.1585	1773.555	1427285.3	1773.15628
1773.15865	1773.15405	1773.354	1324301.3	1447505.3	1773.2675	1400899.3
1448836.3	1773.2705	1773.15716	1773.14773	1773.14972	1773.15669	1773.15472
1773.16085	1773.15349	1773.2976	1773.3192	1773.3416	1427298.3	1423463.3
1773.3514	1773.2937	1773.4859	1400889.3	1324273.3	1773.14798	1773.15326
1423448.3	1773.5689	1773.4848	1773.15325	1773.2449	1773.2914	1773.2977
1397862.3	1773.2801	1773.1588	1773.3304	1773.15012	1773.543	1423501.3
1773.2856	1448666.3	1773.15483	1448667.3	1423489.3	1427283.3	1773.3987
1773.5581	1455312.3	1773.15416	1773.323	1773.5485	1773.15832	1773.5605
1773.5321	1773.15473	1773.3441	1427259.3	1773.5209	1773.5565	1773.5304
1773.14976	1773.15893	1773.2883	1448514.3	1773.5894	1448550.3	1773.2659
1773.15029	1773.1561	1773.5505	1773.5305	1773.547	1448522.3	1773.3277
1400877.3	1408945.4	1773.5395	1448770.3	1773.5277	1773.15398	1421990.3
1773.3434	1448508.3	1773.14747	1773.2592	1427197.3	1773.3239	1448804.3
1773.16099	1773.3388	1421947.3	1397940.3	1438856.3	1773.3327	1773.3014
1773.14942	1773.15234	1773.2917	1773.3009	1773.15694	1773.5027	1773.15474
1324291.3	1447470.3	1773.3425	1773.2823	1773.3456	1773.4849	1773.1497
1773.5474	1773.565	1773.5585	1773.15387	1773.4238	1427241.3	1773.5436
1447495.3	1773.339	1448605.3	1427253.3	1773.15215	1773.3365	1773.14987
1773.15789	1438874.3	1773.3167	1773.15644	1447524.3	1448594.3	1773.541
1773.4256	1773.3405	1423551.3	1773.5195	1773.3373	1397888.3	1408913.4
1773.15064	1773.15916	1773.3773	1773.15797	1773.2588	1773.3352	1773.15153
1773.15505	1773.324	1773.15498	1773.2951	1773.3495	1324262.3	1417028.3
1773.5044	1773.3141	1427318.3	1408971.4	1773.15783	1773.561	1773.2644
1773.4005	1427231.3	1773.5409	1438850.3	1773.16001	1447492.3	1773.2925
1448390.3	1773.14917	1773.2702	1773.15245	1773.15259	1773.15323	1773.15158
1423479.3	1773.5598	1773.15948	1773.15574	1773.299	1773.348	1427277.3
1773.15879	1427287.3	1773.3027	1295805.3	1773.15162	1773.3214	1448797.3

Table 2: Strains that are used for this experiment

1773.3063	1773.15743	1408973.4	1773.3482	1773.15177	1773.5494	1773.15746
1773.539	1295800.3	1773.3219	1773.15248	1773.14734	1427295.3	1423552.3
1773.5158	1773.15947	1448802.3	1773.4251	1773.5275	1422059.3	1448710.3
1448723.3	1354180.3	1773.15894	1773.15542	1773.15799	1773.3005	1408949.4
1773.4217	1773.5064	1773.5444	1773.1602	1773.3295	1773.16083	1773.5603
1324239.3	1773.16093	1448653.3	1773.3361	1773.5264	1427242.3	1773.3281
1773.318	1400885.3	1773.5446	1773.15426	1773.1539	1773.2534	1773.1483
1773.15492	1773.15502	1773.5248	1773.14952	1773.279	1773.336	1773.352
1448831.3	1773.14924	1773.15561	1773.3319	1773.4618	1773.3224	1773.15864
1773.361	1773.1499	1773.2692	1773.5915	1455302.3	1295757.3	1773.3188
1448657.3	1448737.3	1773.2518	1448830.3	1773.5176	1773.2603	1773.15172
1354138.3	1773.2508	1773.2918	1773.592	1773.5553	1773.15262	1773.16012
1773.2676	1773.15613	1773.15365	1324247.3	1773.3016	1773.283	1422024.3
1773.3081	1773.15759	1773.3428	1773.328	1773.15747	1773.15792	1295764.3
1773.3294	1773.2997	1295806.3	1773.5684	1447468.3	1448523.3	1773.5288
1354177.3	1773.297	1773.15424	1773.3263	1773.3577	1422104.3	1773.14771
1448474.3	1773.14862	1773.15484	1773.5562	1773.5112	1773.1514	1773.15256
1773.2438	1773.5438	1773.15093	1773.5519	1773.15846	1421932.3	1773.368
1773.2965	1324287.3	1773.15602	1773.15839	1773.5347	1447458.3	1773.15451
1773.252	1773.2466	1773.15255	1773.2472	1773.15187	1773.2689	1773.2931
1773.2681	1773.15972	1448685.3	1773.15763	1408951.4	1773.5337	1773.14752
1423518.3	1773.5272	1773.3213	1324221.3	1773.15087	1773.5314	1455268.3
1402602.3	1421945.3	1773.4698	1427271.3	1773.267	1448774.3	1773.2936
1397903.3	1773.15612	1773.16021	1773.5357	1773.15878	1438884.3	1773.15099
1773.14997	1408914.4	1773.5402	1448760.3	1773.15703	1773.2544	1773.533
1773.16027	1773.5646	1773.2573	1438846.3	1354134.3	1423548.3	1773.15287
1773.2697	1773.5688	1773.5552	1295774.3	1773.5591	1773.2922	1773.15386
1773.2896	1773.3232	1324238.3	1423470.3	1773.15624	1773.3836	1773.5643
1773.4017	1295760.3	1773.2808	1773.295	1773.319	1773.3323	1773.5727
1773.14928	1448644.3	1448750.3	1773.2576	1773.3344	1773.5065	1773.266
1773.3194	1773.1607	1773.2886	1448466.3	1773.16062	1773.5082	1773.5633
1773.15837	1773.2519	1773.5424	1773.5094	1773.2953	1423495.3	1773.15425
1448460.3	1773.5134	1773.5236	1773.15084	1773.5518	1773.3017	1448449.3
1773.312	1773.5703	1438838.3	1448485.3	1773.15889	1448602.3	1422085.3
1773.5674	1324259.3	1773.15609	1773.5547	1773.3443	1773.3095	1427186.3
1773.15077	1447450.3	1773.5193	1438890.3	1773.5439	1422009.3	1324251.3
1448614.3	1773.3504	1773.15697	1773.15891	1773.1609	1773.15652	1773.3256
1438847.3	1422014.3	1397875.3	1773.15683	1448816.3	1773.15587	1773.3445
1447477.3	1773.3026	1448447.3	1354171.3	1773.2557	1295791.3	1448483.3
1773.15984	1773.2811	1773.5166	1773.5279	1773.3242	1773.345	1773.5482
1773.15363	1773.16063	1427239.3	1773.3049	1354131.3	1773.15183	1773.562
1773.5124	1773.15448	1448792.3	1773.15118	1773.14919	1773.4863	1448410.3

Table 2: Strains that are used for this experiment

1773.154	1773.3453	1773.5299	1448573.3	1773.5653	1400928.3	1773.5269
1773.2837	1324218.3	1773.14925	1773.1495	1773.15196	1773.2546	1773.2626
1448712.3	1773.262	1354151.3	1773.15283	1448785.3	1773.15399	1773.3507
1773.15911	1773.3746	1418251.3	1773.2645	1773.2984	1408904.4	1427258.3
1773.4619	1773.1586	1773.5534	1773.1541	1422107.3	1773.5324	1773.311
1773.3457	1773.14883	1773.2563	1773.5113	1427324.3	1773.15852	1773.3137
1773.2761	1773.2806	1773.5503	1773.16015	1448533.3	1773.15488	1773.1564
1773.5687	1421962.3	1773.5595	1773.15754	1324222.3	1448803.3	1773.5072
1773.5107	1422089.3	1773.3266	1448392.3	1773.15441	1773.566	1773.15115
1773.3481	1773.3494	1773.14858	1397885.3	1773.5271	1773.15678	1773.3018
1773.5654	1427269.3	1773.2734	1773.2871	1402585.3	1397876.3	1448456.3
1773.15083	1773.15781	1773.15929	1773.4239	1448526.3	1773.2805	1773.15548
1773.15554	1773.3143	1773.14841	1773.3389	1422064.3	1354192.3	1773.15226
1773.15779	1773.15269	1773.1489	1773.15313	1773.148	1773.15944	1773.2587
1417008.3	1773.5119	1773.2929	1427190.3	1773.15136	1773.15696	1773.3002
1448775.3	1773.2852	1427309.3	1773.4189	1773.15711	1447526.3	1427240.3
1773.3117	1422095.3	1773.5588	1448618.3	1773.15403	1773.3139	1773.14891
1773.16055	1773.15776	1773.15337	1773.14745	1773.15206	1773.15417	1773.15452
1773.3528	1773.544	1773.5254	1773.3044	1773.5188	1773.5235	1423442.3
1773.5404	1397887.3	1773.3218	1773.3209	1354140.3	1773.14809	1773.15672
1427300.3	1773.2488	1773.5001	1354193.3	1773.5629	1773.5714	1448754.3
1773.14834	1448776.3	1354150.3	1773.15987	1773.3781	1773.502	1324240.3
1447467.3	1773.2487	1773.5146	1448811.3	1773.3191	1773.5057	1773.5031
1417031.3	1773.317	1773.3417	1773.508	1773.15408	1773.5296	1421951.3
1773.15849	1773.2793	1773.3154	1438845.3	1448495.3	1773.2945	1423546.3
1773.1505	1427194.3	1448686.3	1354108.3	1773.14897	1773.15108	1773.5329
1773.3112	1438858.3	1773.14968	1773.15301	1773.3065	1773.15684	1773.4254
1773.15205	1408972.3	1773.16059	1447500.3	1773.15453	1773.288	1773.4237
1455269.3	1773.4242	1773.16041	1773.5636	1773.2782	1447453.3	1773.5315
1773.4128	1773.15778	1773.5892	1773.15586	1773.2913	1773.3099	1773.3342
1773.365	1354146.3	1408975.4	1773.5417	1438853.3	1773.15147	1295765.3
1773.2605	1773.14944	1773.15633	1773.15949	1773.15243	1773.5454	1423474.3
1417021.3	1773.2556	1427212.3	1773.14772	1448768.3	1773.15268	1773.5696
1773.2632	1773.2905	1773.15855	1773.15169	1773.25	1773.14966	1773.1562
1773.3185	1408958.4	1773.14768	1773.2597	1773.14743	1773.1484	1773.15588
1773.4145	1773.2966	1354183.3	1354113.3	1400914.3	1773.15553	1773.5645
1397937.3	1773.15439	1773.302	1447522.3	1773.3096	1773.14794	1773.517
1397896.3	1773.527	1773.507	1773.5502	1773.35	1773.3325	1423571.3
1354178.3	1427254.3	1427208.3	1773.4625	1773.5397	1448561.3	1773.14864
1773.16017	1427265.3	1773.14971	1438864.3	1417015.3	1773.2865	1773.15309
1773.15756	1773.5303	1773.5181	1773.5189	1773.15236	1773.5499	1773.2536
1773.3149	1448801.3	1773.5476	1773.15748	1267361.3	1773.15095	1773.5222

Table 2: Strains that are used for this experiment

1455287.3	1448475.3	1773.3448	1773.15688	1773.1485	1773.15819	1773.2476
1773.2585	1447475.3	1773.15104	1773.4213	1423462.3	1408950.4	1427291.3
1397932.3	1773.15955	1773.3774	1773.5097	1773.2717	1448396.3	1773.2746
1773.5943	1423511.3	1773.15288	1773.5223	1773.2927	1773.15175	1773.347
1408953.4	1448813.3	1773.15125	1773.15103	1773.1547	1448519.3	1773.5511
1773.15854	1773.4203	1773.5698	1773.511	1447517.3	1773.16092	1773.286
1448708.3	1773.15597	1773.3322	1397894.3	1773.14874	1773.14902	1773.15185
1773.352	1773.2956	1324268.3	1773.5418	1422055.3	1773.15432	1773.15635
1773.5431	1773.2841	1448835.3	1773.316	1448838.3	1354161.3	1423537.3
1773.1574	1773.15818	1773.4199	1448661.3	1448426.3	1773.14918	1773.5004
1773.15537	1773.3068	1773.15292	1408931.4	1448761.3	1773.1606	1773.5919
1773.15285	1400902.3	1773.15438	1773.15559	1773.259	1773.327	1773.15239
1773.5374	1773.5074	1773.15225	1397892.3	1773.3286	1354189.3	1773.14786
1773.3588	1773.14901	1773.15677	1773.15028	1773.5628	1773.15253	1773.2906
1773.485	1427195.3	1773.3173	1773.14896	1773.5721	1448706.3	1773.15327
1773.5559	1773.2832	1773.16042	1324246.3	1773.15543	1773.2443	1773.5273
1773.15503	1773.5673	1773.15202	1773.5358	1773.2666	1773.15738	1773.5586
1423527.3	1448739.3	1773.15341	1773.15354	1773.2738	1448701.3	1773.2545
1773.15137	1773.15324	1773.5276	1448574.3	1773.2962	1773.5655	1773.321
1773.14995	1417014.3	1773.2651	1773.5086	1438862.3	1421971.3	1773.2988
1773.15946	1773.15558	1773.15076	1448742.3	1448693.3	1773.5922	1295723.3
1448450.3	1448534.4	1773.15757	1773.293	1773.15402	1438872.3	1427182.3
1773.16023	1773.2493	1447446.3	1773.3085	1438848.3	1773.2875	1773.14728
1295720.3	1773.15116	1773.2892	1438866.3	1773.14981	1773.15072	1773.16049
1447519.3	1773.15556	1417019.3	1773.4028	1448762.3	1448619.3	1773.4628
1773.5197	1773.15027	1773.3278	1773.2577	1773.16064	1324227.3	1773.5117
1773.15967	1773.15152	1773.3196	1773.5058	1773.15887	1427191.3	1448638.3
1773.201	1773.15518	1448601.3	1773.15535	1773.3	1427306.3	1773.5523
1773.15908	1773.2572	1448646.3	1773.5343	1773.2575	1773.298	1773.15216
1773.3492	1773.4204	1397912.3	1773.15499	1773.2526	1773.4224	1773.15737
1773.3427	1773.5899	1397907.3	1773.1563	1773.2478	1773.15692	1773.2561
1773.3046	1773.15803	1421975.3	1773.15808	1773.15922	1448481.3	1773.15926
1773.149	1773.5526	1324256.3	1427211.3	1324223.3	1773.257	1773.3078
1773.5411	1773.3395	1773.15132	1773.5443	1417012.3	1773.15258	1773.15541
1773.1503	1448719.3	1773.3362	1773.15914	1773.2855	1455273.3	1773.5641
1773.2785	1773.15378	1773.2482	1421982.3	1324283.3	1408925.4	1773.2934
1773.276	1773.3317	1773.15978	1773.4163	1773.2667	1773.16003	1423469.3
1447472.3	1773.15204	1773.289	1773.4626	1324292.3	1773.5623	1427255.3
1773.2648	1773.2598	1397921.3	1423477.3	1773.2862	1773.4257	1773.2713
1773.15621	1773.15917	1773.15646	1773.5075	1773.5676	1418250.3	1773.14854
1773.5394	1448591.3	1773.14964	1773.1552	1448432.3	1773.2693	1773.3032
1773.14939	1448772.3	1448781.3	1773.542	1773.5006	1773.15411	1773.3261

Table 2: Strains that are used for this experiment

1773.287	1773.15009	1773.5708	1448763.3	1773.265	1773.2963	1773.4172
1773.2961	1773.16082	1422018.3	1773.366	1422045.3	1773.2602	1448834.3
1773.294	1427217.3	1423426.3	1773.1496	1773.15176	1773.5203	1773.5391
1423557.3	1773.15982	1773.16098	1773.2532	1773.15302	1423543.3	1773.16051
1773.159	1295758.3	1773.2978	1773.14969	1773.4086	1773.3374	1773.5129
1773.512	1448454.3	1324266.3	1773.15166	1773.15266	1427238.3	1455305.3
1773.153	1773.15355	1773.2537	1773.14825	1773.2665	1773.1501	1773.15845
1773.202	1773.3128	1773.33	1773.15625	1773.3236	1773.5359	1773.5423
1773.15681	1773.4853	1773.5	1773.14848	1773.3189	1773.15075	1773.15211
1773.14851	1773.5262	1773.5306	1418254.3	1773.3403	1773.14982	1773.331
1773.2529	1773.277	1422044.3	1773.14833	1773.2933	1448692.3	1773.14965
1773.2617	1448837.3	1773.14967	1773.5243	1773.14778	1773.5318	1773.2594
1773.5528	1773.5533	1427248.3	1773.5367	1438861.3	1773.5435	1773.15049
1773.3039	1324289.3	1324258.3	1773.295	1447523.3	1773.16011	1773.5634
1773.14905	1773.3067	1773.15068	1773.15433	1773.1592	1448670.3	1773.3479
1773.2654	1447507.3	1773.5225	1447440.3	1773.2646	1773.2615	1773.3669
1773.5372	1773.2538	1773.3532	1423574.3	1773.5619	1773.15953	1773.15968
1773.14955	1423509.3	1773.3146	1773.5265	1773.1474	1438887.3	1427177.3
1422082.3	1447484.3	1354157.3	1773.4407	1773.5609	1400875.3	1448629.3
1423505.3	1773.264	1773.3093	1773.15815	1773.15035	1773.366	1773.5386
1773.3273	1427244.3	1773.3275	1773.16078			
