# Supplementary material for: Machine learning and phylogenetic analysis allow for predicting antibiotic resistance in M. tuberculosis
Source: BMC Microbiol. 2023 Dec 20;23:404. doi: 10.1186/s12866-023-03147-7 (PMC10731705; doi:10.1186/s12866-023-03147-7)
Supplement: Supplementary file 1 — Additional file 1. [file 12866_2023_3147_MOESM1_ESM.zip › Supplement_5.pdf]

Supplement 5

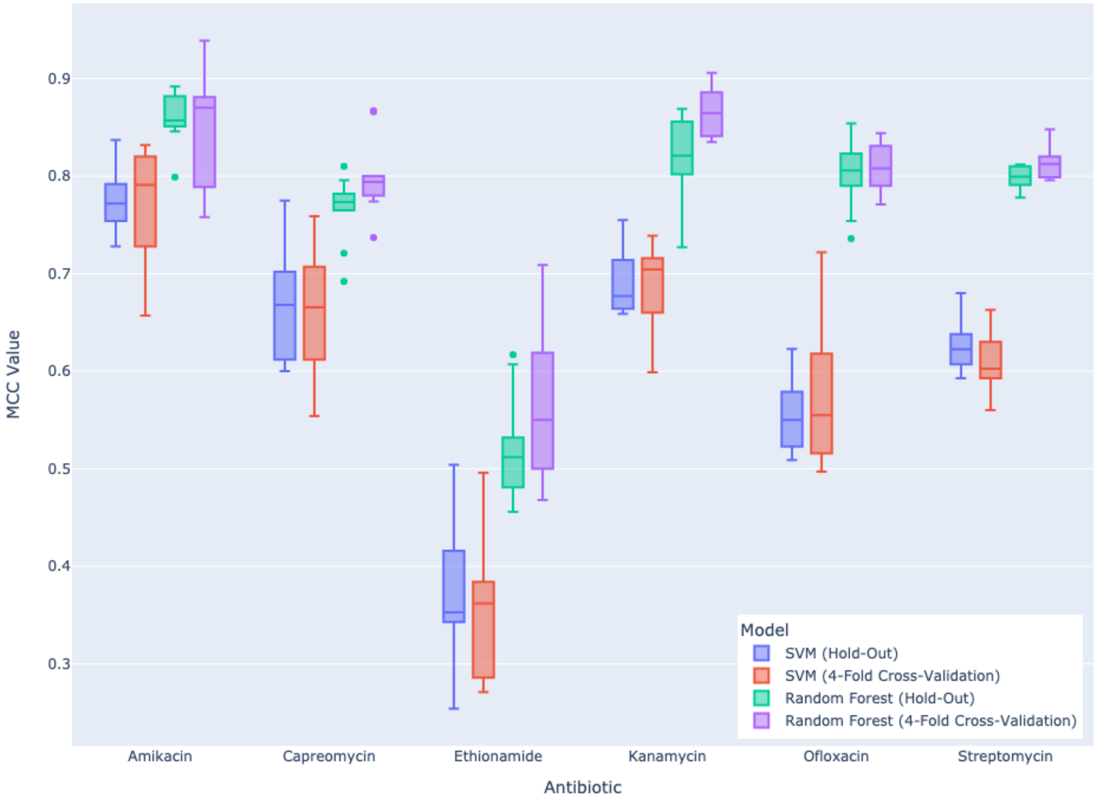

Fig. 1: MCC values of SVM & RF models trained with random train test split and randomized seeds

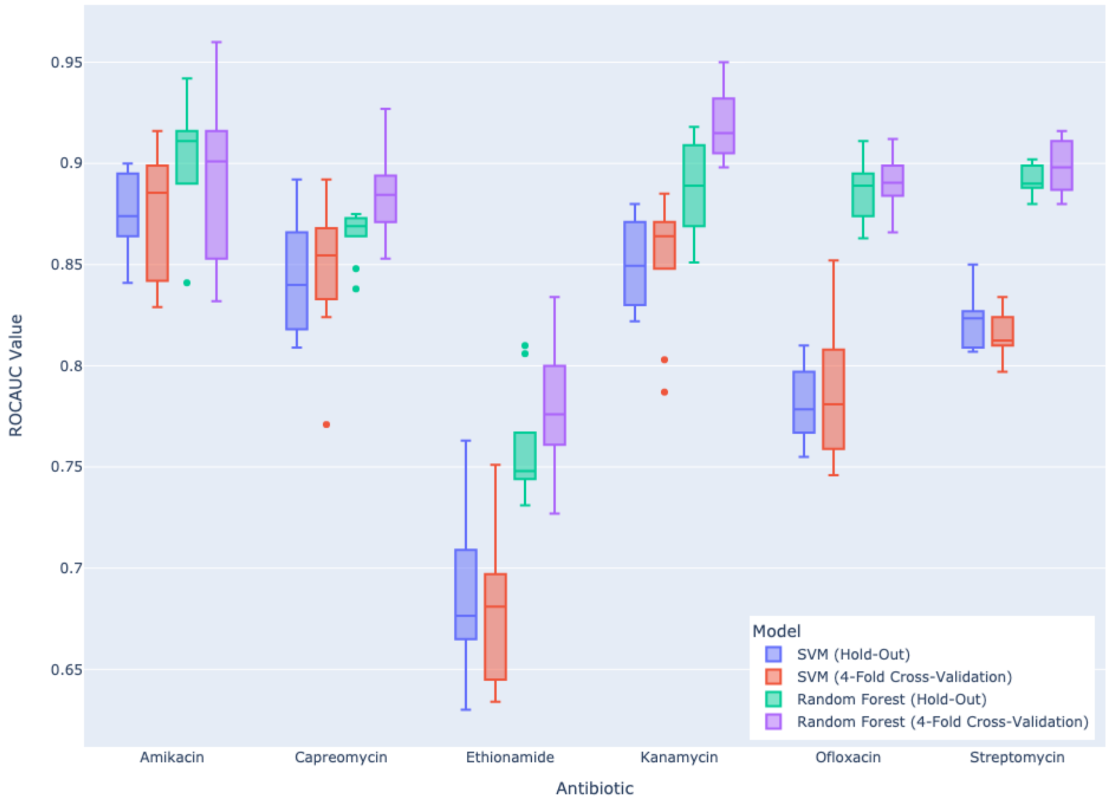

Fig. 2: ROCAUC values of SVM & RF models trained with random train test split and randomized seeds
